# Tropical carbon sink accelerated by symbiotic dinitrogen fixation

**DOI:** 10.1038/s41467-019-13656-7

**Published:** 2019-12-10

**Authors:** Jennifer H. Levy-Varon, Sarah A. Batterman, David Medvigy, Xiangtao Xu, Jefferson S. Hall, Michiel van Breugel, Lars O. Hedin

**Affiliations:** 1 Princeton Environmental Institute, Princeton University, Princeton, NJ 08544 USA; 2 Department of Ecology and Evolutionary Biology, Princeton University, Princeton, NJ 08544 USA; 3 School of Geography and Priestley International Centre for Climate, University of Leeds, Leeds, LS2 9JT UK; 4 Smithsonian Tropical Research Institute, Balboa, Ancón, Panamá Panama; 5 Cary Institute of Ecosystem Studies, Millbrook, NY 12545 USA; 6 Department of Geosciences, Princeton University, Princeton, NJ 08544 USA; 7 Department of Biological Sciences, University of Notre Dame, Notre Dame, Indiana 6556 USA; 8 Department of Ecology and Evolutionary Biology, Cornell University, Ithaca, NY 14850 USA; 9 ForestGEO, Smithsonian Tropical Research Institute, Balboa, Ancón, Panamá Panama; 10 Yale-NUS College, Singapore and Department of Biological Sciences, National University of Singapore, Singapore, Singapore

**Keywords:** Element cycles, Ecological modelling, Carbon cycle, Forest ecology

## Abstract

A major uncertainty in the land carbon cycle is whether symbiotic nitrogen fixation acts to enhance the tropical forest carbon sink. Nitrogen-fixing trees can supply vital quantities of the growth-limiting nutrient nitrogen, but the extent to which the resulting carbon–nitrogen feedback safeguards ecosystem carbon sequestration remains unclear. We combine (i) field observations from 112 plots spanning 300 years of succession in Panamanian tropical forests, and (ii) a new model that resolves nitrogen and light competition at the scale of individual trees. Fixation doubled carbon accumulation in early succession and enhanced total carbon in mature forests by ~10% (~12MgC ha^−1^) through two mechanisms: (i) a direct fixation effect on tree growth, and (ii) an indirect effect on the successional sequence of non-fixing trees. We estimate that including nitrogen-fixing trees in Neotropical reforestation projects could safeguard the sequestration of 6.7 Gt CO_2_ over the next 20 years. Our results highlight the connection between functional diversity of plant communities and the critical ecosystem service of carbon sequestration for mitigating climate change.

## Abstract (web summary)

The contribution of symbiotic dinitrogen fixation to the forest carbon sink could change throughout forest succession. Here the authors model nitrogen cycling and light competition between trees based on data from Panamanian forest plots, showing that fixation contributes substantially to the carbon sink in early successional stages.

## Introduction

Tropical forests offer a crucial service for limiting global warming and mitigating climate change. Mature tropical forests store large amounts of carbon^1,2^, while young forests—either recovering from disturbance or established as reforestation projects—can sequester substantial quantities of atmospheric CO_2_ in forest landscapes^1,3^. It is increasingly appreciated that reforestation of previously deforested and degraded lands can be a significant management tool for global carbon sequestration^4^. For example, the Bonn Challenge^5^ is an international effort to reforest up to 350 million hectares of land by 2030. The success of this and similar efforts depend crucially upon understanding the factors that govern the rate at which carbon is taken up and stored in re-growing forests.

Nitrogen fixation may provide tropical forests with the nitrogen they need as they rapidly build biomass following disturbance. For example, nitrogen-fixing trees supplied ~50% of the nitrogen needed to support forest recovery in the first few decades of secondary succession in Panamanian forests^6^. Whether this applies uniformly across tropical forests is an open question, and it is plausible that between-forest differences in populations of nitrogen-fixing trees contribute to the dramatically different rates by which tropical forests appear to recover from disturbance^7,8^. This issue is both fundamental to our understanding of the tropical carbon cycle and of practical importance for the capture of CO_2_ through reforestation^3^.

Moreover, different assumptions about how fixation is represented in global biogeochemical models can dramatically influence predictions about global carbon and/or nitrogen dynamics^9–11^. Of particular concern is whether models are structured in a way that captures the local-scale feedback between nitrogen fixation and the recovery of carbon pools following forest disturbance events^12–14^.

It is difficult, however, to directly measure the role of nitrogen-fixing trees in the forest carbon cycle. First, a carbon–nitrogen fixation feedback would impact forest carbon stocks over the course of forest succession, such that quantification in nature would require monitoring over decades to centuries. Second, manipulative field experiments cannot quantify the influence of fixation on forest carbon uptake, as there is no known method for inhibiting fixation or removing nitrogen-fixing trees without also perturbing the ecosystem. Third, by recycling fixed nitrogen, fixers can influence neighboring non-fixing trees, but this indirect effect is difficult to isolate and quantify in nature.

Here we evaluate the impact of the carbon–nitrogen fixation feedback by combining field observations across 300 years of succession in Panamanian tropical forests with a land biogeochemistry–vegetation model, ED2, designed to capture plant–soil–nutrient interactions at the scale of individual trees. We demonstrate that nitrogen fixation enhances the tropical carbon sink through a feedback with carbon accumulation, and that the strength of this effect depends on individual-scale competition between nitrogen fixing and non-fixing trees in the forest community. The carbon sink effect is particularly strong in young forests following disturbance, and declines gradually as forests mature.

## Results

### Modeling approach

The ED2 model was developed from ref. ^15^ and is subjected to sensitivity analyses in Supplementary Note 3, in Supplementary Figs. 3–11, and in previous publications^16–20^. ED2 resolves plant–plant competition, plant–soil nutrient cycling, and nitrogen fixation in an individual’s local environment. Thus, the model can capture the essential interactions that scale up from individuals to the carbon–nitrogen feedback dynamics at the ecosystem level. This individual-based underpinning (Methods) differs fundamentally from the traditional practice (reviewed in ref. ^11^) of scaling fixation to ecosystem-level properties including evaporation, primary production, or average concentrations of soil nutrients within a forest.

Based on field observations from Panama^6,21,22^, we developed a nitrogen-fixing plant functional type (PFT) that possessed the ability to down-regulate fixation when soil nitrogen does not limit plant growth (Table 1). This PFT was added to an existing set of three PFTs that have conventionally been used by ED2 to simulate tropical forests, including: (i) an early successional non-fixing PFT characterized by rapid growth, high mortality, low wood density, and high leaf nitrogen; (ii) a mid-successional non-fixing PFT, with medium growth, mortality, wood density, and leaf nitrogen; and (iii) a late-successional non-fixing PFT characterized by low growth and mortality, high wood density, and low leaf nitrogen (Methods; Table 1).

**Table 1:** Ecophysiological and life-history traits of the plant functional types (PFTs) used in model simulations.

Trait	Units	Plant functional types				
		Early-successional	Mid-successional	Late-successional	Fixer with fixation ability	Fixer without fixation ability
Leaf C:N	weight ratio	15.3	28.3	61.3	17.6	17.6
Specific leaf area	m² kg C^−1^	21.7	17.2	15.1	19.7	19.7
Wood density	g cm^−3^	0.40	0.60	0.87	0.60	0.60
Photosynthetic capacity per unit leaf area	µmol m^−2^ s^−1^	22.5	15.0	7.5	32.4	32.4
Density independent mortality	yr ^−1^	0.081	0.054	0.018	0.066	0.066
Maximum N_2_ fixation rate	g N fixed kg biomass^−1^ day ^−1^	0	0	0	0.39	0

We evaluated our model against >13,000 trees identified to species across a well-characterized chronosequence of tropical lowland forests (5, 12, 30, 80, and ~300 years following disturbance; Methods)^6^. We sought to minimize parameter tuning by basing initial conditions and our fixation parameterization on field observations from our Panamanian study forests (Supplementary Tables 1–4; Methods). We calibrated two critical parameters by adjusting: (i) the maximum leaf carboxylation across PFTs to match the initial (<30 years) rate of forest biomass accumulation, and (ii) the rate of forest gap creation to match the observed steady-state (300 years) biomass. A parameter sensitivity analysis is presented in Supplementary Note 3.

### Model—data comparison

**Fig. 1:**
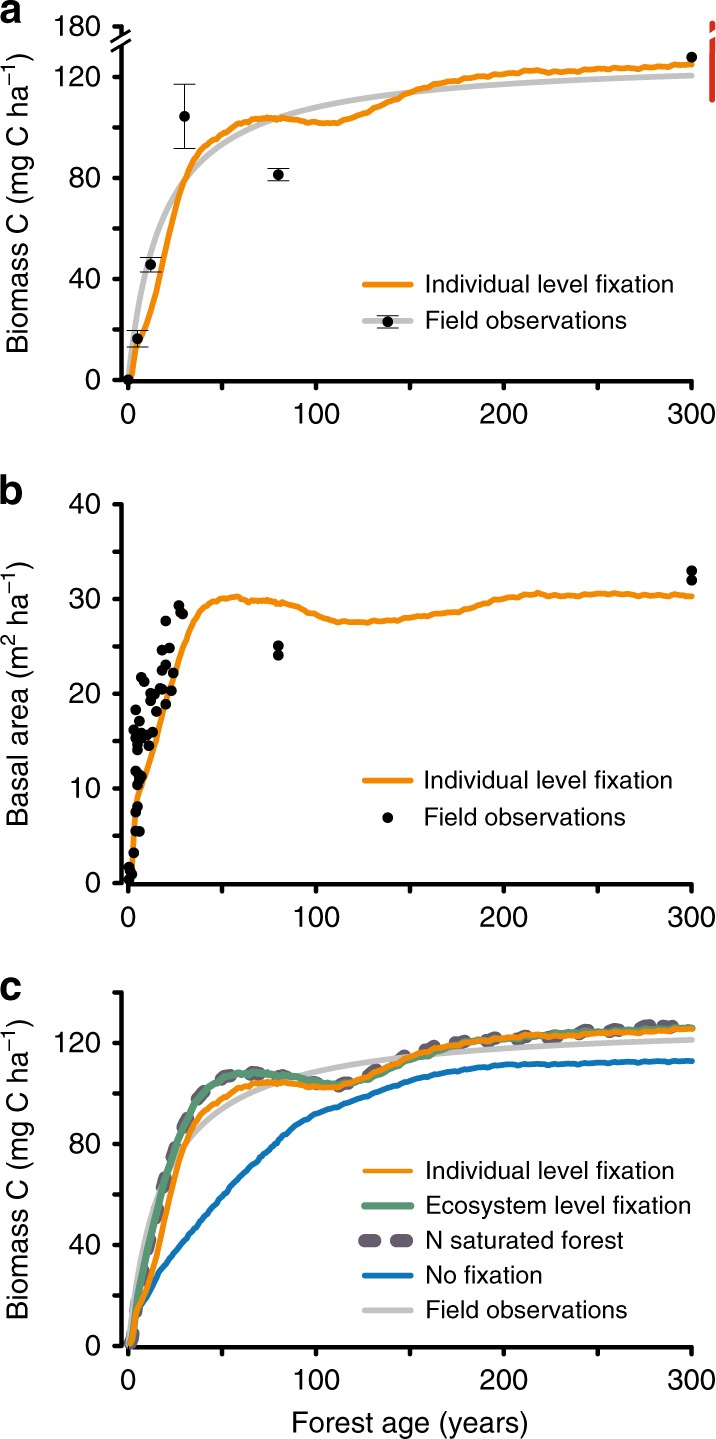
Successional dynamics in tropical rainforests. **a** Tree biomass carbon accumulation (above + belowground; Mg C ha^−1^) over successional age (years since disturbance) observed across our Panamanian forests. Black dots indicate field observations^6^ of mean biomass carbon ± standard error of the mean (SEM) for *n* = 4 plots per year for forests 5–30 years and *n* = 2 plots per year for forests 80–300 years. Gray line represents Michaelis–Menten curve fitted to field observations^6^. Orange line represents our model predictions for forests that include all plant functional types and nitrogen fixation. Red line shows biomass observed in 11 additional mature forests in Panama^6, 38^. **b** Ecosystem pattern of sum of tree basal area (m^2^ ha^−1^) over forest successional age observed^21^ across our Panamanian forests (black points; each point represents one forest plot) and predicted by our model that includes all plant functional types and nitrogen fixation (orange line). **c** Tree biomass carbon accumulation (above + belowground; Mg C ha^−1^) observed across our Panamanian forests (gray line; as described in **a**) and predicted by our individual level model for forests with (orange line) and without (blue line) nitrogen fixation. Also shown are predictions from our ecosystem-level (green line) and nitrogen-saturated (dashed gray line) models. The nitrogen-saturated simulation is initiated with high levels of soil N (20 kg Nm^−2^) and does not allow dissolved organic nitrogen, dissolved inorganic nitrogen, or nitrogen gas to be lost from the ecosystem. Both the individual level and ecosystem level fixation simulations are initiated with realistic soil N levels (0.05 kg Nm^−2^) and allow for nitrogen losses from the ecosystem.

**Fig. 2:**
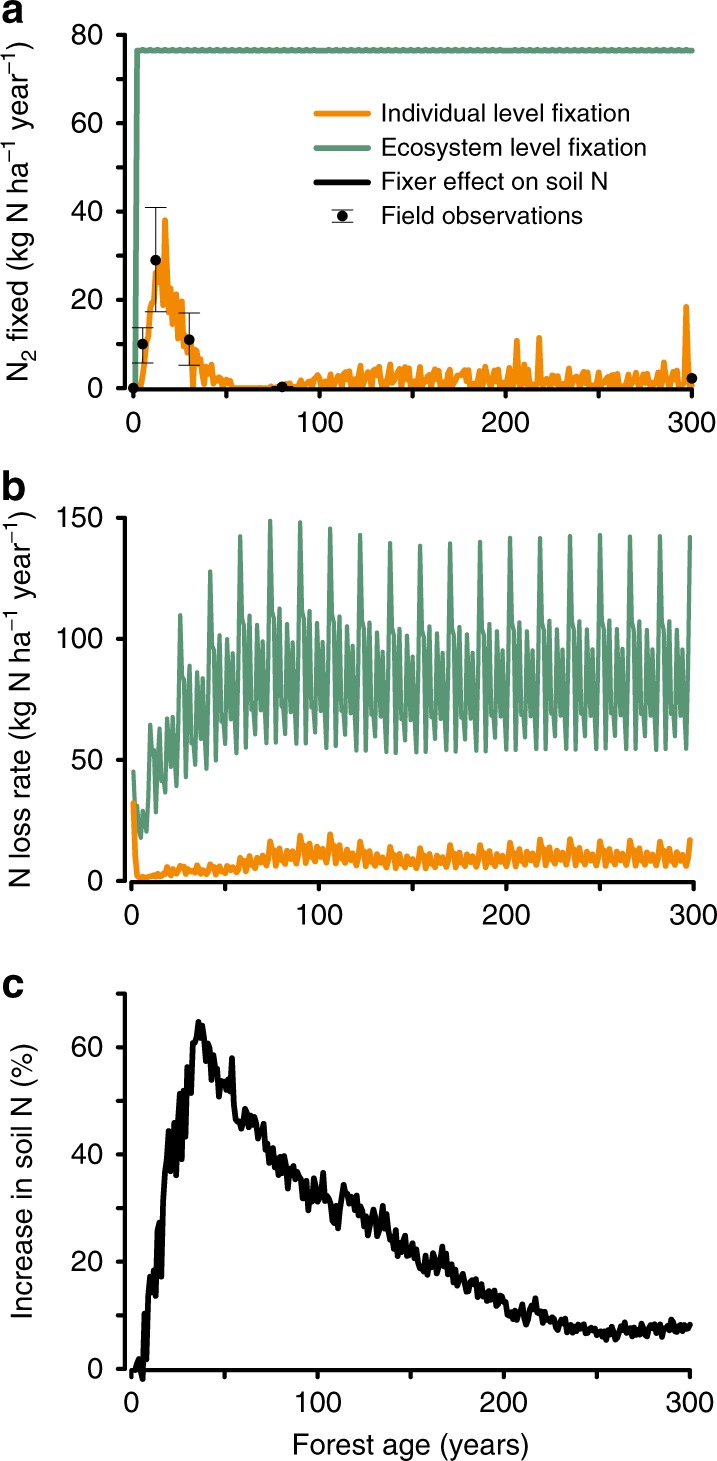
Nitrogen cycle in tropical rainforests recovering from land use. **a** Symbiotic nitrogen fixation (kg N ha^−1^ yr^−1^) over forest successional age (years) observed in Panama^6^ (black points represent mean ± SEM for *n* = 4 plots per year for forests 5–30 years and *n* = 2 plots per year for forests 80–300 years) and predicted by our model for forests that include all plant functional types and nitrogen fixation regulated at the individual plant level (individual level fixation; orange line) or fixation scaled to an ecosystem property, such as forest evapotranspiration (ecosystem level fixation; green line). **b** The nitrogen loss rate (kg N ha^−1^ yr^−1^) over forest successional age (years) predicted by our model for forests with individual (orange line) or ecosystem-level fixation (green line), as described in (**a**). **c** The increase in soil nitrogen (N; %) predicted by our model for forests that include all plant functional types and individual level fixation compared to forests without fixation. The increase in soil nitrogen was calculated from model predictions of forests with all plant function types and fixation minus forests with all plant functional types and no fixation. For all model scenarios, the pattern of variation in nitrogen fluxes reflect our use of a looped 16-year meteorological dataset from ASP (Methods).

Our model closely re-created the most essential carbon and nitrogen dynamics observed at the ecosystem scale and over successional time (Figs.1 and 2). The rate of plant carbon accumulation was highest in the first few decades of recovery, progressively slowed, and saturated at ~120 Mg C ha^−1^ of total biomass in mature forests (Fig. 1a). In parallel, forest basal area (summed across trees) increased dramatically in early succession and saturated at ~300 years, again closely following field observations (Fig. 1b).

The lack of plots between 80 and 300 years, and the difficulty of dating mature forests (Methods), introduces some uncertainty about the biomass saturation value for mature forests. Comparison against 11 mature Panamanian forests^6,21^ (red bar, Fig. 1a) shows that the biomass in this study’s mature plots is at the low end of other forests. Post-disturbance nitrogen demand would be even higher in those forests, indicating that the results presented here likely are conservative.

Crucially, our model predicted the complex pattern of fixation observed over successional time (Fig. 2a, orange line with field observations^6^ indicated by closed circles): a rapid increase in fixation in early succession, peaking at ~40 kg N ha^−1^ yr^−1^, and then dropping to low levels (~2 kg N ha^−1^ yr^−1^) as forests matured. This pattern is consistent with the idea that trees fix the most in the first decades of forest recovery, when nitrogen limitation is most severe and plant nitrogen demand is large relative to soil nitrogen supply. As the net accumulation of biomass slows down in mature forests, however, fixation drops to low levels. In mature forests, fixation by trees is maintained by forest gap disturbances, in which re-growing trees cause high rates of net biomass accretion—thus creating small-scale pockets of transient nitrogen limitation. Nitrogen fixers populate these pockets and increase both carbon and nitrogen input to the forest.

### Effect of fixation on forest development

We compared our default model setup against a modified simulation in which we turned off the capacity of trees to fix nitrogen. However, we included a PFT in which all traits were identical to the fixer PFT except it could not fix nitrogen (Table 1); this allowed us to evaluate the impact of nitrogen fixation independent of any other trait that characterized the fixer PFT.

Our results show that inclusion of the fixation trait had both short- and long-term effects on forest development (Fig. 1c; orange vs. blue lines). First, biomass accumulated more rapidly in the first half-century of succession than in forests without fixation. Second, biomass equilibrated at a ~10% higher level in late succession than in forests without fixation. We next address how these trends were linked to the forest nitrogen cycle, and then examine their consequences for forest carbon storage.

### Effect of fixation on the nitrogen cycle

Forests with the fixation trait accumulated soil nitrogen more rapidly in the first half-century of recovery, but this effect decreased as trees began to down-regulate fixation over the course of succession (Fig. 2c; Fig. 1c orange vs. blue lines). Forests that lacked the fixation trait still built up an internal nitrogen cycle over successional time due to retention of external inputs from nitrogen deposition, but at a rate substantially slower than forests with fixation. As observed for biomass, a ~10% increase in nitrogen accumulation remained even in mature forests. This sustained nitrogen effect was caused by individual trees that up-regulated fixation in the nitrogen-limited conditions created in local forest gaps. We discuss the carbon consequences of these dynamics further below.

### Plant functional type dynamics and fixation

**Fig. 3:**
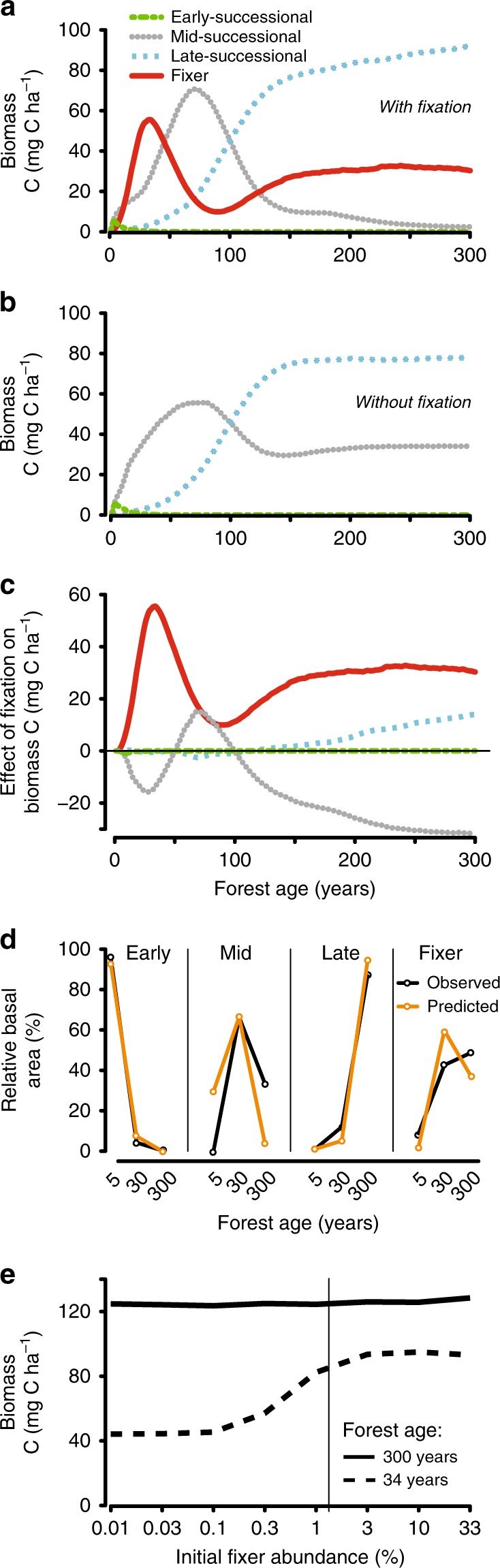
Plant functional types and biomass carbon in tropical rainforests recovering from land use. **a**, **b** Carbon (above + belowground) dynamics (Mg C ha^−1^) of different plant functional types (early-, mid-, and late-successional and fixers) over forest successional age (years) predicted by our model for forests that include all plant functional types with fixation (**a**) and forests that include all plant functional types but with the fixation trait turned off (**b**). Lines represent each plant functional type as described in the legend in **a**. **c** Decoupling the direct effect and indirect effect of nitrogen fixation on plant carbon accumulation (%) throughout tropical forest succession (years). The effects were calculated as the difference in biomass carbon for the fixers (direct fixation effect, red line) and the non-fixing PFTs (indirect fixation effect, gray, blue, and green lines) between model predictions of forests with all plant functional types with fixation vs. forests with all plant functional types without fixation. Supplementary Table 4 and Methods provide details. **d** Comparison of basal area at each forest age relative to the total basal area of each plant functional type observed across forests at 5, 30, and 300 years of recovery from land use in Panama (black lines) and predicted by our model (orange lines). The field observations were calculated for the five most abundant species at each forest age summed across *n* = 4 plots for ages 5 and 30 and *n* = 2 plots for age 300 years. Source data are provided in Supplementary Note 4. **e** The effect of initial fixer abundance (% basal area) in the forest community on plant biomass carbon (above + belowground; solid lines; Mg C ha^−1^) in forests of 34 (dashed line) and 300 (solid line) years. The vertical line indicates the initial fixer abundance used for all model simulations in Figs. 1, 2, and 3 a–d.

Our model broadly re-created the observed successional changes in forest community PFT composition (Fig. 3). We compared model predictions against five dominant tree species at each stand age, selected to represent the successional stage of each community (Methods). These species combined occupied 42% of total basal area in 5-year forests, 25% in 30-year forests, and 30% in the 300-year forest.

Our model predicted a shift from early, to mid, and to late-successional PFTs as forests recovered from disturbance (Fig. 3a), matching the overall and highly dynamical pattern observed in nature (Fig. 3d). The predicted sequence of PFT replacement largely reflected density-dependent mortality caused by competition for nitrogen and light: The early successional PFT was most competitive immediately following disturbance, as high leaf nitrogen and rapid growth rate allowed it to colonize soil before plant-available nitrogen became limiting.

Within a few years, the early succession PFT was out-competed by the fixer PFT once forest nitrogen demand exceeded the supply from soil mineralization or deposition input. Our model predicted peak fixer abundance close to the period in which the observed fixation was highest (Fig. 2a vs. Fig. 1a), but also that fixers began to lose their competitive edge as fixation progressively improved supplies of soil nitrogen. As a result, fixers began to down-regulate fixation (to reduce the carbon cost of acquiring nitrogen). Over time, fixers were increasingly out-competed by the mid-successional PFT, which could grow more efficiently in non-nitrogen limited conditions owing to the intermediate nitrogen content of its biomass.

Finally, low nitrogen content made the late-successional PFT most competitive in the mature forest phase, even though low leaf nitrogen is physiologically associated with low rates of photosynthesis and plant growth. The diversity of PFTs in our modeled mature forests was maintained by gap-phase dynamics: local disturbance events continuously re-created the competitive interactions between PFT’s and allowed the nitrogen-fixing PFT to remain abundant in the forest canopy and sub-canopy.

In contrast, modeling the forest without the fixation trait resulted in a much different successional PFT sequence (Fig. 3b), with persistently lower soil nitrogen stores (Fig. 2c) causing the mid-successional PFT to remain competitively stronger than the fixer PFT (without fixation ability) throughout succession. As a result, the abundance of carbon-rich late-successional trees and forest biomass was depressed (discussed below).

These community-level results support the theory that fixers play a critical role in both the nitrogen biogeochemistry and PFT interactions of tropical forests, consistent with the following observations:^6,14,21–25^ (i) fixers out-compete non-fixers in early succession, when soil nitrogen limits plant growth; (ii) fixers can down-regulate or switch off fixation as the ecosystem nitrogen cycle develops and nitrogen no longer limits plant growth; and (iii) fixers can persist in the canopy of mature forests without actively fixing nitrogen, but can up-regulate fixation in low-nitrogen patches created by tree-fall gaps.

### Representation of nitrogen fixation in models

We next compared our individual-based model against the traditional approach of scaling nitrogen fixation to a local ecosystem-level property, typically evapotranspiration (ET) or net primary production (NPP) (Table 1 in refs. ^11^ and^26–28^). We used the common (5 of 9 models in ref. ^11^) scheme of scaling fixation to ET (Methods) but our analysis applies in principle also to NPP or other schemes scaled to aggregate ecosystem properties. This alternative ecosystem-based approach: (i) could not capture the observed up- vs. down-regulation of fixation and the nitrogen cycle over successional time (Fig. 2a, b), (ii) greatly over-predicted nitrogen fixation overall, and (iii) generated unrealistically large ecosystem nitrogen losses compared to measures of dissolved (e.g., 6–10 kg N ha^−1^ yr^−1^ in 6 Costa Rican forests;^29^ 1.4–7.2 in four Hawaiian forests;^30^ and 2.6–2.7 in two Chinese forests^31^) or gaseous (0–9 kg N ha^−1^ yr^−1^ in 12 Hawaiian forests;^30,32^ and 5.6–15 in 2 Chinese forests^31^) nitrogen losses across unpolluted tropical forests.

We infer that the representation of nitrogen fixation at scales of individual tree physiology, and the local feedback between individual tree fixation and soil conditions, is critical for resolving when and where nitrogen-fixing trees deploy fixation, as well as the dynamics of the forest nitrogen cycle throughout succession (Fig. 2; Supplementary Note 1).

### Effect of nitrogen fixation on carbon accumulation

We next performed model experiments to evaluate whether fixation can enhance forest carbon storage through a carbon–nitrogen feedback. Our individual-based model suggested that in the 30 years following disturbance, forests with abundances of nitrogen-fixing trees similar to our field sites accumulated carbon at nearly twice the rate of forests in which fixation was absent (Fig. 1c, orange vs. blue line). Fixation thus caused the additional storage of ~40 Mg C ha^−1^ at 34 years compared to the no-fixation scenario, but this carbon benefit declined as nitrogen limitation began to wane in older forests (discussed below). However, mature forests with fixation indefinitely stored ~10% more carbon (~12 Mg C ha^−1^) than no-fixation forests due to (i) new nitrogen fixed within forest disturbance gaps (as discussed above), and (ii) the indirect effect of nitrogen fixation on PFT competition during forest recovery (discussed below).

Comparison of the Fig. 1c carbon accumulation curves further show that, consistent with our Panama field observations^6^, nitrogen fixers in the individual level model were capable of supplying enough new nitrogen to maintain weak but transient nitrogen limitation on carbon accumulation in the first 60 years of succession (c.f., orange line of individual model vs. dashed gray line of nitrogen-replete conditions). The ecosystem-level model did not re-create these transient dynamics, but, instead, wholly alleviated nitrogen limitation on forest growth throughout succession (c.f., green line of ecosystem model vs. dashed gray line). While different parameter choices could reduce nitrogen fixation in the ecosystem model, it could not introduce the feedback dynamics necessary to re-create the observed up- and down-regulation of fixation or the transient nitrogen limitation on carbon accumulation over succession.

### Direct and indirect effects of fixation

We next evaluate the mechanisms by which fixation influenced forest carbon accumulation in the individual-based model. We can distinguish two independent causes: a direct effect due to the growth of nitrogen-fixing trees vs. an indirect effect due to the influence of fixation on the growth of non-fixing trees (mediated by either soil nitrogen fertilization or plant–plant competition). We distinguished these mechanisms by comparing carbon accumulation in two modeled forests with identical initial species composition, allowing the nitrogen-fixing PFT to fix nitrogen in one but not the other.

Our results show that the direct fixation effect on forest carbon storage was greatest in early succession when fixers grew and fixed most (~55 Mg C ha^−1^ at ~35 years; Fig. 3c, red line), and after a mid-successional dip, again became significant in mature forests (~30 Mg C ha^−1^). The trend resulted from the disproportionately rapid growth of fixers in the nitrogen-limited conditions of early succession, and a sustained enhancement of forest biomass caused by fixation associated with the gap-phase disturbance dynamics of mid and late succession.

We next evaluated the indirect carbon effect caused by the N subsidy provided by the fixer PFT in our model scenarios. First, by building up the nitrogen cycle and by sharing nitrogen with neighboring trees, the presence of trees with the fixation trait triggered increased growth and competitive success of the late-successional plant functional type (Fig. 3c, blue line). Crucially, this effect depends on the sustained creation of gap disturbances in mature forests, within which fixers maintain fixation following these localized disturbances. This effect caused forest carbon to gradually increase over successional time, up to a peak of ~10 Mg C ha^−1^ in the mature forest.

Second, despite increased ecosystem nitrogen, fixers suppressed the growth and accumulation of carbon by mid-successional tree species by up to ~30 Mg C ha^−1^ in the mature forest (Fig. 3c, gray line). This suppression arose mainly from plant–plant competitive dynamics in the high light, low nitrogen conditions of tree-fall gaps.

Our results indicate that nitrogen fixation has a complex effect on the forest carbon balance, with both positive and negative components. Combining both direct and indirect effects, fixation had an overall net positive effect, resulting in an 85% short-term increase (at 35 years of recovery), and a 10% long-term increase in carbon storage of the mature forest (Fig. 1c, orange vs. blue lines).

### Effect of fixer abundance

Finally, we examined whether the initial abundance of nitrogen-fixing trees determined the ability of forests to serve as a carbon sink. We varied the percent of all trees that were fixers in the establishment stage of forest succession. Our results identify a 3% basal area threshold, below which fixers could not increase in abundance rapidly enough to safeguard fixation and carbon accumulation over the course of succession. Above this threshold, however, increased fixer abundance did not translate into increased carbon storage. This tipping point was apparent at ~34 years of successional age, but was largely unimportant in mature forests at 300 years as, over time, atmospheric deposition could supply the required nitrogen  (Fig. 3e; dashed vs. solid line). We infer that the density of fixer seedlings in the initial stage of forest establishment is critical for their impact on the forest carbon cycle over the first several decades of succession.

## Discussion

Overall, our findings demonstrate that nitrogen fixation can be a critical determinant of the capacity of tropical forests to serve as sustained carbon sinks through two mechanisms: (i) by alleviating nutrient limitation in a local plant–soil feedback; and, (ii) by changing the competitive dynamics and sequence of the tree community and/or PFTs during succession. The dynamics of these mechanisms could only be captured when we modeled fixation at the individual tree and PFT scale. Scaling fixation to an aggregate ecosystem property failed to resolve critical dynamics in our Panamanian forests including: successional down-regulation of the nitrogen cycle, an emergent carbon–nitrogen feedback tied to local disturbances, and the indirect influence of fixation and tree community composition on the sequence of PFT succession.

Our analysis of fixer-rich tree communities in Panamanian forests suggest that fixation can be sufficient to maintain rapid carbon accumulation following disturbance and/or to respond to increasing atmospheric CO_2_. However, abundances and activities of fixers differ substantially across tropical forests worldwide^23,33^, with likely implications for the extent to which fixation can safeguard carbon accumulation. Moreover, it should be remembered that other factors can limit both nitrogen fixation and tree growth in tropical forests, most notably soil phosphorus and molybdenum (co-factor in the nitrogenase enzyme)^23^. Our focus on nitrogen fixation stems from its central role in structuring the nitrogen cycle and carbon–nitrogen interactions in many tropical forests.

Our finding of a fixer initial abundance threshold suggests that efforts to predict and manage carbon accumulation in tropical forests must account for the density-dependent effect of fixers on the nitrogen cycle and on competition between PFTs over successional time. Tree species and/or PFT inventories may be needed to predict the geographical potential for additional carbon storage. Reforestation efforts might consider optimal plantings of nitrogen-fixing trees, above the threshold at which we would expect that the forest carbon cycle is insensitive to fixer abundances.

How can our results inform the development of models for the tropical forest biome? Most central is perhaps our finding that carbon accumulation can be closely linked to the abundance and activities of nitrogen fixers, which, in turn, depend on the dynamics of the tree community over succession and across geographical regions. The common approach of scaling fixation to an aggregate ecosystem property cannot capture individual- or community-level interactions. Instead, promising avenues forward may include the development of simplified frameworks for PFT competition, or the derivation of fixer abundance and/or functional thresholds to safeguard carbon accumulation.

We used a back-of-the-envelope calculation to evaluate whether our results might be relevant for understanding the tropical carbon sink. By combining our model plus field-based observations with global analyses of Neotropical forest carbon trends^8^, we estimate that nitrogen-fixing trees could theoretically increase carbon capture in the reforestation projects pledged under the Bonn Challenge^5^ by an additional 1.8 Gt C (equivalent to 6.7 Gt CO_2_) over the next 20 years (Supplementary Note 2).

It is more difficult to evaluate the potential impact of fixation on carbon uptake by extant mature Neotropical forests (i.e., excluding forests recovering from recent human disturbance). Our study identifies a specific mechanism by which fixation can enhance mature forest carbon stores, but the comparison of the model against field observations was limited by the lack of plots between 80 and 300+ years of age. However, our model may underestimate the carbon benefit of fixation in mature forests, since biomass in our forests was at the low end of the range reported from 11 other mature Panamanian forests^6,21^. If we apply our modeled carbon benefit to extant mature Neotropical forests, we estimate^5,34,35^ that fixation may have enabled the storage of an additional 10 PgC (~37 Gt CO_2_) compared to forests absent fixation (Supplementary Note 2). This large value begs for further study of this potential mechanism, and how it may vary as a function of abundances of nitrogen-fixing trees and other local factors (e.g., soil molybdenum and/or phosphorus^23^, or frequency of disturbance^25^).

Our model and field observations have broad implications for understanding tropical forests. Differences in fixer abundances between individual forests and across biogeographical regions^23,33^ likely impacts the carbon sink and may be key for understanding limits to future carbon uptake in recovering and mature forests. The potential for indirect effects of nitrogen fixers on non-fixing species raises new questions about determinants of the tropical carbon sink, including whether fixation is critical for the establishment and rapid growth of carbon-rich late successional trees.

## Methods

### Model overview

ED2 is a dynamic vegetation model that also simulates biogeochemical cycles and the exchanges of carbon, water, radiation, and energy between the land and atmosphere^15^. The model solves a system of partial differential equations to describe the behavior of a vertically stratified, spatially distributed collection of cohorts of individual plants^20^. The system of equations enables the model to: (i) track gap-scale changes in the biophysical, ecological, and biogeochemical structure of the ecosystems; (ii) incorporate the spatially localized competition between plant cohorts; (iii) clearly distinguish between cohorts with different nitrogen fixation strategies; and (iv) capture the impacts of subgrid-scale disturbances on the structure and function of the ecosystem. Further details on the structure of the model are provided in ref. ^15,36^, and its application to tropical forests has been described in ref. ^16–19^.

We tailored the model to a tropical forest that includes PFTs of early-, mid-, and late-successional plants and nitrogen fixers, representing a continuum of successional life history strategies^17,20,36,37^. Of these, only the early-, mid-, and late-successional PFTs have been presented in earlier work that used ED and ED2^16–19^ As in those studies, early successional trees have higher photosynthetic capacity and low wood density, enabling them to grow quickly under high light conditions, but at the expense of reduced growth and increased mortality under low-light conditions. Conversely, late successional trees have lower photosynthetic capacity and higher wood density, which causes them to grow more slowly than early successional trees under high incoming radiation, but their lower maintenance costs allow them to outperform early successional trees under low-light conditions. Mid-successional trees have intermediate trait values, resulting in performance characteristics that are also intermediate between those of early- and late-successional trees. The PFT trait values used here (Table 1) are within the range of those previously used for ED and ED2, which have in fact varied somewhat across studies^16–18,20,37^. Here, we develop an open nitrogen cycle as a new component of the model (Supplementary Fig. 2; Supplementary Tables 1–4).

We compared this individual level model against the common^11,26–28^ approach of scaling fixation to the ecosystem-level property of evapotransporation. In all ecosystem-level simulations, we estimated fixation following equation no. 5 in ref. ^27^ and did not include a carbon cost. The fixed nitrogen was added directly into the bioavailable soil nitrogen.

### The nitrogen cycle

The nitrogen cycle developed in this study includes: (1) belowground competition for nitrogen, (2) leaf nitrogen resorption, (3) plant nitrogen uptake, (4) nitrogen limitation on photosynthesis, (5) nitrogen limitation on reproduction, (6) a nitrogen-fixing PFT, (7) facultative symbiotic biological nitrogen fixation, (8) hydrologic losses of dissolved organic and inorganic nitrogen, (9) gaseous nitrogen loss, and (10) nitrogen deposition. All carbon and nitrogen dynamics are calculated on a daily time step except for recruitment and wood growth rates, which are updated monthly. See Supplementary Methods for details.

### Field site and model evaluation

We used data from the Agua Salud Project (ASP, https://forestgeo.si.edu/research-programs/affiliated-programs/agua-salud-project) in Panamanian lowland tropical moist forest to evaluate our model. ASP includes a 300-year chronosequence of land-use recovery from pasture. We use the 300 years plots to approximate mature forest conditions; these plots have not had any pasture and likely no to minimal human disturbance. Analyses of successional trajectories in plant carbon, PFT dynamics, and nitrogen fixation were based on our previous study^6,21^ of 16 forest plots at 5, 12, 30, 80 years, and near maturity (~300 years old; *n* = 4 0.2 ha plots for ages 5–30 and *n* = 2 1.0 ha plots for ages 80 and 300). Other measurements (forest basal area and initial tree species composition) included observations from up to 112 ASP plots of varying chronosequence age and size (0.1–1.0 ha). ASP receives ~2700 mm of rainfall yr^−1^ with a dry season from mid-December to April. See refs. ^6,22,38,39^ for additional details. Field observations^6,21,22^ of plant carbon accumulation, nitrogen fixation, and forest basal area were used to evaluate ED2. The observed community pattern of relative basal area was determined from the five dominant species at each forest age (5, 30, and 300 years) and assigned as early- (dominants at 5 years; occupying 42% of total stand basal area), mid- (dominants at 30 years; 25% basal area) and late- (dominants at 300 years; 30% basal area) successional PFTs for comparison to model PFTs. For each species at each time point (5, 30, 300), the ratio of basal area to total basal area of that species across all years was multiplied by 100. The fixer PFT was calculated the same way except that the basal area of all fixer species was considered since species capable of fixation are known^6,24,40^.

### Sensitivity analysis

Several sensitivity analyses have been performed with respect to ED2 simulations of tropical forests. These have focused on phenology, soil hydrology, and external climate forcing^18,35,38^. Here, we perform a sensitivity analysis focusing on parameters directly related to nitrogen fixation and/or that were subject to tuning. See Supplementary Note 3 for details.

### Model initialization

We initialized the model in several ways depending on the simulation. The initial PFT composition for the individual level fixation simulation was determined by analyzing ASP demography data from the 5-year-old plot censuses. We determined the wood density and plant functional type for every tree using a global database^41,42^ and field observations^39^. If wood density information was not available for a species, we assigned an average wood density based on genus and then family. The initial abundance of each PFT in the model matched the abundance of each PFT at the 5-year-old ASP plots: early successional, mid-, late- and fixers comprised 0.26, 0.87, 0.23, and 0.016 individuals m^−2^ respectively, totaling 1.38 individuals m^−2^.

For all other simulations, we kept constant both the total tree density (1.381 individuals m^−2^) and the density distribution of early-, mid-, and late-successional PFTs. Soil nitrogen was initialized at 0.05 kg N m^−2^_,_ with 70% distributed evenly between the slow and structural nitrogen pools that decompose slowly, 20% in the fast soil nitrogen pool (e.g., leaf litter), and 10% in the bioavailable soil nitrogen pool.

The distinctive characteristics of each PFT are in Table 1 and additional details for the fixer PFT are included in Supplementary Methods. Supplementary Table 4 summarizes the PFT combinations used to initialize each simulation.

### Meteorology in the model

We used a 16-year meteorological dataset based on in situ climate observations at Barro Colorado Island (~5 miles from ASP) available through the Smithsonian Tropical Research Institutes’ Physical Monitoring Program (http://biogeodb.stri.si.edu/physical_monitoring/). This dataset was compiled for 1997–2012, outliers were removed, and missing data were replaced with climate data from ref. ^43^. The 16-year dataset was looped over the 300-year simulation.

### Calculations of the direct and indirect effects of fixation

We determined the direct and indirect effects of fixation on the forest aboveground carbon sink by evaluating two different model scenarios: (1) the simulation with all PFTs including nitrogen fixation, and (2) the simulation with all PFTs absent fixation (Supplementary Table 4). This allowed us to calculate the direct fixation effect as the difference in biomass of the nitrogen-fixing PFT between the scenarios. The indirect effect then could be calculated as the total carbon difference between the two runs minus the direct fixer effect. Finally, we decomposed the indirect effect into contributions by the early, mid, vs. late-successional PFTs (calculated as the contribution of each PFT to the total indirect carbon effect).

### Reporting summary

Further information on research design is available in the Nature Research Reporting Summary linked to this article.

## Supplementary information

Supplementary Information

Reporting Summary

## Data Availability

Field data in Figs. 1–2 is from ref. ^6,21^. Field data in Fig. 3d is available in Supplementary Note 4.

## Source Data

Source Data

## Code availability

The code for the ED2 model used in this publication is available in the GitHub repository https://github.com/davidmedvigy/Levy-Varon-etal. The Supplementary Methods provides details on variables and parameters used to create the model.
