# Supplementary material for: Tropical carbon sink accelerated by symbiotic dinitrogen fixation
Source: Nat Commun. 2019 Dec 10;10:5637. doi: 10.1038/s41467-019-13656-7 (PMC6904724; doi:10.1038/s41467-019-13656-7)
Supplement: Supplementary file 1 — Supplementary Information [file 41467_2019_13656_MOESM1_ESM.pdf]

Tropical carbon sink accelerated by symbiotic dinitrogen fixation

Levy-Varon et al.

Supplementary Information

Tropical carbon sink accelerated by symbiotic dinitrogen fixation

Jennifer H. Levy-Varon, Sarah A. Batterman, David Medvigy, Xiangtao Xu,
Jefferson S. Hall, Michiel van Breugel, and Lars O. Hedin

The supplementary information contains the following sections:

Supplementary Methods: Model description for ED2.

Supplementary Note 1: Evaluation of nitrogen limitation in the individual and ecosystem-level fixation simulations.

Supplementary Note 2: Scaling up the fixation effect to Neotropical forests.

Supplementary Note 3: Sensitivity analysis of model to parameter values for the nitrogen fixer PFT and disturbance.

Supplementary Note 4: Calculation in support of Fig. 3d.

Supplementary Figure 1. Comparison of soil N in simulations with and without nitrogen fixation.

Supplementary Figure 2. Diagram of the nitrogen cycle in ED2.

Supplementary Figure 3. Sensitivity of plant biomass, basal area and N2 fixed to the maximum specific N_2 fixation rate.

Supplementary Figure 4. Sensitivity of plant biomass (a), basal area (b), and N_2 fixed (c) to the maximum carboxylation rate of the N-fixer PFT.

Supplementary Figure 5. Effect of N_2 fixation on biomass for different values of the maximum carboxylation rate.

Supplementary Figure 6. Sensitivity of simulated plant biomass, basal area, and N_2 fixed to the canopy gap creation rate.

Supplementary Figure 7. Effect of N_2 fixation on biomass for different values of the gap creation rate.

Supplementary Figure 8. Sensitivity of simulated plant biomass, basal area, and N_2 fixed to the inorganic N loss factor.

Supplementary Figure 9. Effect of N_2 fixation on biomass for different values of the inorganic N loss factor.

Supplementary Figure 10. Sensitivity of simulated plant biomass, basal area, and N_2 fixed to the maximum N storage capacity.
Supplementary Figure 11. Effect of N_2 fixation on biomass for different values of the maximum N storage capacity.
Supplementary Table 1. Model parameter values and definitions for the nitrogen cycle in ED2.
Supplementary Table 2. Model equations for the plant nitrogen dynamics in ED2.
Supplementary Table 3. Model equations for soil nitrogen dynamics.
Supplementary Table 4. Summary of model simulations and plant functional type (PFT) composition.
Supplementary References.

Supplementary Methods: Model description for ED2

A. Overview

The ED2 model simulates processes across a wide range of time scales[1]. Land-atmosphere exchange of carbon, water, radiation, and energy and soil hydrology are updated on a dynamic time step typically of the order of seconds. Soil biogeochemistry, including carbon (C) and nitrogen (N), is updated daily. Vegetation dynamics and competition between plant functional types (PFTs) are updated monthly. Previous studies have presented the model structure[1-2], its previous application to tropical forests[1,3-6] and sensitivity analyses[3-4]. We here give a brief overview of the model, and then in the subsequent sections give detailed descriptions of model components.

A dynamic timestep (~seconds) is used to compute land-atmosphere exchanges of carbon, water, radiation and energy. The vertical profile of photosynthesis is computed using the Farquhar et al. (1980)[7] model of photosynthesis coupled to the Leuning (1995)[8] stomatal conductance scheme. The model uses a two-stream approximation to compute vertical transfers of direct and diffuse photosynthetically-active radiation and near infrared radiation, as well as transfers of longwave radiation. Heat and moisture are transferred between ~20 soil layers[9]. A canopy air space, acting as a reservoir of heat, moisture and CO_2, also exists between the overlying atmosphere and the soil. Water can accumulate on vegetation through interception of precipitation and dew formation, and each vegetation layer is able to carry out transpiration. Root and stem respiration are also computed on the sub-daily time step.

Vegetation dynamics are updated on a monthly time step. Vegetation is described in terms of the number density of trees of a given plant functional type and size existing in a patch of a given age. The patch age corresponds to the time since last disturbance. A set of diagnostic equations is used to resolve plant height, diameter, active biomass pools (leaves, fine roots), a storage pool, and a wood biomass pool. The different vegetation biomass pools have prescribed, PFT-dependent C:N ratios. The model computes rates of growth, mortality, recruitment and disturbance, and then uses these rates to solve a system of size- and age-structured partial differential equations[2]. These equations are able to closely approximate the first moment of the equations of a corresponding stochastic gap model, enabling the model to explicitly track the changing landscape age-structure and associated sub-grid scale heterogeneity arising from prior disturbance history[10].

Spatial heterogeneity arises from disturbance events. Forest gaps arise when trees die (modeled here as mortality) and are represented by a newly-formed patch of ground that has a seed bank and low amounts of soil nutrients but no established trees. Within-patch heterogeneity of resources is created vertically by a light gradient within canopy layers and a water gradient within soil layers. Horizontal within-patch heterogeneity for resources like soil nitrogen are not explicitly represented in the model; however, horizontal heterogeneity is created from a plant's perspective by including in the model allocation of resources based on between-tree competition that results from the plant's physical and physiological characteristics, as determined by the PFT and growth of the plant. For example, as described below in Section C, within-patch heterogeneity of soil nitrogen is simulated by allowing trees differential access to soil nitrogen based on their root biomass. Small trees that have small root sytems will have access to less mineralized nitrogen than large trees that have large root systems. For more information on how the physical and physiological differences of PFTs impact tree competition and utilization of resources, see ref.10. The biogeochemistry module governing nitrogen transformations is run on a daily time step. An overview of the model's new nitrogen cycle is shown in Supplementary Figure 3. The features include: 1) belowground plant competition for nitrogen; 2) leaf nitrogen resorption; 3) nitrogen limitation on photosynthesis; 4) nitrogen limitation on plant reproduction; 5) a nitrogen-fixing PFT; 6) facultative symbiotic biological N_2 fixation; 7) hydrologic losses of dissolved organic and inorganic nitrogen (DON and DIN); 8) gaseous nitrogen loss; and, 9) nitrogen deposition. For further details, see Sections B, C, D and E below.

B. Nitrogen-fixing PFT

In this paper, we present a new nitrogen-fixing PFT with parameters constrained by tropical forest measurements. In tropical forests, field and experimental observations suggest that facultative biological nitrogen fixation is a dominant strategy for N_2 fixers[11-15]. Our nitrogen-fixing PFT was therefore designed to have the ability to up- and down-regulate fixation depending on the nitrogen economy of individual plants. The wood density, specific leaf area, and leaf C:N of the nitrogen-fixing PFT are based on field measurements from a tropical forest in Costa Rica[16] and Panama[11,17]. In accordance with field observations, fixers have a higher maximum carboxylation rate then other PFTs[18] and have a higher foliar nitrogen concentrations than non-fixers[19-23]. Mortality rate is a function of wood density and increased by 20% for the fixer PFT, reflecting our hypothesis that the nutrient-rich leaves of the N_2 fixer population increases herbivory and consequently mortality (Table 1). The remaining elements of the fixer PFT parameterization are identical to early successional non-fixer PFT.

C. Individual-level plant nitrogen dynamics

Changes in an individual plant's nitrogen budget are computed daily as the balance between plant nitrogen uptake and plant nitrogen losses. Consistent with the ED2 model's representation of ecosystem structure and composition, plant N uptake and loss rates are computed for each cohort of plants of PFT *i* and size *z*, existing in a patch of landscape of age *a*, at time *t*. A plant's daily N uptake is calculated as the mininmum of the nitrogen required to maintain its stoichiometry and the nitrogen available (from the soil and biological nitrogen fixation). On daily timescales, plants lose N through the turnover of leaves and fine roots.

In what follows, we will present calculations that are used to predict daily N uptake. These calculations include: 1) plant N demand for growth ($N_{demand,growth}$; kg N day^{-1}; Supplementary Equation 1); 2) plant nitrogen wanted for storage ($N_{want,storage}$; kg N day^{-1}; Supplementary Equations 2-4); 3) plant available nitrogen from soil ($N_{avail,soil}$; kg N day^{-1} ; Supplementary Equation 5); 4) plant nitrogen acquired through soil uptake ($N_{plant,up,soil}$; kg N day^{-1}; Supplementary Equation 6); and, 5) plant nitrogen acquired through biological nitrogen fixation (BNF; kg N day^{-1}; Supplementary Equations 7-9). We combine Supplementary Equations 6 and 9 to calculate daily N uptake ($N_{plant,up}$; kg N day^{-1}; Supplementary Equation 10).

Finally, we present plant nitrogen loss ($N_{daily,plant,loss}$; kg N day^{-1}; Supplementary Equation 11) and internal recycling of N through resorption ($N_{resorption}$; kg N day^{-1}; Supplementary Equation 12). All symbols are explained in Supplementary Table 1.

Plant nitrogen demand for growth ($N_{demand,growth}$; kg N day^{-1}) is calculated as the product of the plant C:N ratio and the carbon available for growth (Supplementary Equation 1).

$$N_{\text{demand,growth}}(i,z,a,t) = \sum_j \frac{1}{(C:N)_j(i)} \left(\frac{dC_j(i,z,a,t)}{dt} \right) \tag{1}$$

Here, the summation over j is over plant tissues (leaves, fine roots, wood, reproductive structures) and calculates the plant C:N. The amount of carbon available for growth that will be allocated to tissue j is represented by C_j. The carbon available for growth is calculated by the standard ED2 equations for photosynthesis, respiration, and allocation[1].

Plant nitrogen required for storage ($N_{want,storage}$; kg N day^{-1}) and the nitrogen storage reservoir ($N_{storage}$ kg N). Unlike previous versions of the model, we include a storage reservoir of N that is decoupled from carbon. This storage reservoir can be filled each day unil a prescribed maximum is reached. The nitrogen required to fill the storage reservoir ($N_{want,storage}$) is calculated daily as the difference between the maximum amount of nitrogen allowed in storage ($N_{max,storage}$; kg N) and the amount of nitrogen currently in storage ($N_{storage}$) (Supplementary Equation 2). At the beginning of the simulation and when recruits are created, the storage reservoir is initialized with 0 kg N plant^{-1}.

$$N_{\text{want,storage}}(i,z,a,t) = \frac{N_{\text{max,storage}}(i,z,a,t) - N_{\text{storage}}(i,z,a,t)}{\tau} \tag{2}$$

The maximum amount of nitrogen allowed in storage (Supplementary Equation 3) equals 1.33 times the nitrogen in the leaves of a fully flushed crown, and therefore increases with tree maturity.

$$N_{\text{max,storage}}(i,z,a,t) = 1.33 \frac{C_{\text{crown}}(i,z,a,t)}{(C:N)_{\text{leaf}}(i)} \tag{3}$$

Here, C_{crown}(kg C) is the carbon stored in a full crown, and is calculated using plant allometry described in ref. 1. $N_{max,storage}$ is based on observations of nitrogen being remobilized within trees for the seasonal growth of leaves. Isotope studies show that the proportion of N remobilized from storage for new growth can vary greatly among species ranging from 9-100% of N stores[24].

Given that most plants store more N than they need to produce the first flush of leaves for seasonal growth, we allow plants to exceed the N needed to support the growth of a full crown and take a conservative estimate of storing an additional 33% of the crown N.

As the tree grows, nitrogen accumulates each day in the storage pool until it is used for reproduction ($N_{\text{reproduction}}$)(Supplementary Equation 13). Nitrogen entering the storage pool comes from the N resorbed during leaf turnover ($N_{\text{resoprtion}}$; kg N day^{-1}) (Supplementary Equation 12), and nitrogen from the available soil N ($N_{\text{avail,soil}}$; kg N) (Supplementary Equation 5) after nitrogen uptake for growth occurs[25]. Therefore, the change in the N_{storage} pool is updated daily according to:

$$\frac{dN_{\text{storage}}(i,z,a,t)}{dt} = N_{\text{resorption}}(i,z,a,t) - N_{\text{reproduction}}(i,z,a,t) + \min\left(N_{\text{want,storage}}(i,z,a,t), \left(\frac{N_{\text{avail,soil}}(i,z,a,t)}{\tau}, N_{\text{demand,growth}}(i,z,a,t)\right)\right)$$
(4)

Next, we calculate the nitrogen available to the plant from the soil and from biological nitrogen fixation. Since fixation is an energetically costly process, fixation will only occur if there is some residual N demand after uptake for growth from the soil is considered.

Plant available nitrogen from soil ($N_{\text{avail,soil}}$; kg N) (Supplementary Equation 5) and belowground competition for nitrogen. The soil nitrogen available to each plant is the product of the mineralized N in the patch ($N_{\min}$; kg N m^{-2})(Supplementary Equation 15) and the proportion of the plant's fine root biomass to the total fine root biomass in the patch.

$$N_{\text{avail,soil}}(i,z,a,t) = \frac{C_{\text{root}}(i,z,a,t)}{\sum_{i,z} C_{\text{root}}(i,z,a,t)} * N_{\min}(a,t) \quad (5)$$

Here, C_{root} (kg C) is the amount of C in fine roots for an individual plant and the summation is over all trees existing in the patch of landscape (kg C m^{-2}). By allowing differential access to soil nitrogen based on root biomass, belowground competition for soil nitrogen is represented. Small trees which have small root sytems will have access to less mineralized nitrogen than large trees which have large root systems.

Combining Supplementary Equations 1, 2 and 5, we obtain a plant's **daily N uptake from soil ($N_{\text{plant,up,soil}}$; kg N day^{-1}) (Supplementary Equation 6)** as:

$$N_{\text{plant,up,soil}}(i,z,a,t) = \min\left(N_{\text{demand,growth}}(i,z,a,t) + N_{\text{want,storage}}(i,z,a,t), \frac{N_{\text{avail,soil}}(i,z,a,t)}{\tau}\right) \quad (6)$$

A plant's daily N uptake from the soil is the mininmum of the nitrogen wanted for growth ($N_{\text{demand,growth}}$) (Supplementary Equation 1) and storage ($N_{\text{want,storage}}$) (Supplementary Equation 2) or the nitrogen available from the soil ($N_{\text{avail,soil}}$) (Supplementary Equation 5) that day (t).

Plant nitrogen acquired through biological nitrogen fixation (BNF; kg N day^{-1}). Biological nitrogen fixation is based on the amount of nitrogen the plant would like to fix and the amount of nitrogen it can actually fix when fixation rate is constrained using field observations. We first calculate the plant demand for BNF and then the maximum plant BNF.

The amount of nitrogen the plant would ideally like to fix, **biological nitrogen fixation wanted** (BNF_{want}; **kg N day^{-1})** is calculated according to:

$$\text{BNF}_{\text{want}}(i,z,a,t) = \frac{\left(N_{\text{demand,growth}}(i,z,a,t) - \frac{N_{\text{avail,soil}}(i,z,a,t)}{\tau}\right) * (C{:}N)_{\text{leaf}}(i)}{\text{BNF}_{\text{cost}} + (C{:}N)_{\text{leaf}}(i)} \quad (7)$$

The numerator represents the potential gain in carbon from N fixation without considering a carbon cost. Here, we first take the difference between the nitrogen that could be used for growth ($N_{\text{demand,growth}}$) (Supplementary Equation 1) and the nitrogen available to the plant from the soil ($N_{\text{avail,soil}}$) (Supplementary Equation 5) that day (t). This difference gives the nitrogen that could be used for growth if the plant can obtain it through biological nitrogen fixation. We convert this value to units of carbon by multiplying by $(C{:}N)_{\text{leaf}}$. The denominator calculates the carbon needed to grow leaves with the $(C{:}N)_{\text{leaf}}$. Here, we add the C cost per kg of fixed N **(BNF_{cost}; 9.12 kg C kg^{-1} N)** to the *C:N* of the leaf tissue receiving the fixed N. Taken together, the equation optimizes the fixation demand and provides the amount of nitrogen that could be used by the plant for growth after the carbon cost of fixation is considered. The carbon cost of BNF is based on Gutschick et al. (1981)[26], which accounts for the metabolic cost of fixation (12 kg glucose kg^{-1} N) plus a cost for building and maintaining root nodules and the N_2 fixation mechanism (10.8 kg glucose kg^{-1} N).

The maximum amount of biological nitrogen fixation (BNF_{max}; kg N day^{-1}) is constrained using a field observed relationship between tree biomass and fixation rate. BNF_{max} is calculated as:

$$\mathrm{BNF}_{\mathrm{max}}(i,z,a,t) = \mathrm{Biomass}(i,z,a,t) * \mathrm{BNF}_{\mathrm{rate}}$$

(8)

Where Biomass includes both above and belowground biomass (kg biomass) and the BNF_{rate} = 0.0039 kg N fixed kg biomass^{-1} day^{-1} for the fixer PFT (based on the maximum rate observed from > 250 trees[11]). For early-, mid-,late- and null fixer PFTs BNF_{rate} = 0 g N fixed kg biomass^{-1} day^{-1}. This relationship allows fixation capacity to increase with tree maturity for N_2 fixers. Additionally, because the total amount of nitrogen acquired by a plant is rate constrained, N_2 fixers can still experience nutrient limitation.

Finally, the actual amount of nitrogen fixed through biological nitrogen fixation **BNF (BNF; kg N day^{-1})(Supplementary Equation 9)** equals the minimum of either the demand for BNF (BNF_{want})(Supplementary Equation 7) or the maximum amount of fixation possible in a day (BNF_{max})(Supplementary Equation 8).

$$\mathrm{BNF}(i,z,a,t) = \min\left(\mathrm{BNF}_{\mathrm{want}}(i,z,a,t), \mathrm{BNF}_{\mathrm{max}}(i,z,a,t)\right) \quad (9)$$

Combining equations 6 and 8 we obtain a plant's total **N uptake (kg N day^{-1})(10)** as:

$$N_{\mathrm{plant,up}}(i,z,a,t) = N_{\mathrm{plant,up,soil}}(i,z,a,t) + \mathrm{BNF}(i,z,a,t) \quad (10)$$

A plant's daily N uptake is the nitrogen acquired from the soil $N_{plant,up,soil}$ (Supplementary Equation 6) and the nitrogen acquired through biological nitrogen fixation (BNF)(Supplementary Equation 9).

Allocation of N from the soil and BNF. Nitrogen taken up from the soil is allocated first to the growth of plant tissues ($N_{want,growth}$). Any remaining soil N available to the plant is then allocated to nitrogen storage. Currently, the model does not include a carbon cost associated with acquiring N from the soil. A recent study suggests that implementing a cost for N that is acquired through nutrient-mining microbes such as mycorrhizal fungi could further improve model predictions[27] and this process would be one of the next steps for future work. Finally, fixation

occurs only if there is some residual N demand after uptake from the soil is considered. The fixed N will only be used for the growth of plant tissues, but not to fill the $N_{storage}$ reservoir.

Plant nitrogen loss and N resorption. Plant N losses occur on two time scales, daily and monthly. **Turnover of leaves and fine roots ($N_{daily,plant,loss}$; kg N day^{-1})** occur daily:

$$N_{\text{daily,plant,loss}}(i, z, a, t) = \frac{\alpha_{\text{leaf}(i)} C_{\text{leaf}}(i,z,a,t)}{(C{:}N)_{\text{leaf}}(i)} + \frac{\alpha_{\text{root}(i)} C_{\text{root}}(i,z,a,t)}{(C{:}N)_{\text{root}}(i)} \quad (11)$$

The intrinisic turnover times of leaves and fine roots are α_{leaf} and α_{root}, respectively[1]. In (Supplementary Equation 11), we convert the carbon being lost from the plant through leaf and root turnover (numerators) to the nitrogen lost from this process by dividing by the PFT-specific *C:N* ratio for each structure (denominators). Before leaf abscission, 48% of N is resorbed from leaf tissue (a_{leaf}) and moved to the nitrogen storage pool[25,28] as long as the $N_{storage}$ capacity has not been reached. We can therefore calculate the **N resorbed during leaf turnover ($N_{resorption}$; kg N day^{-1})** as:

$$N_{\text{resorption}}(i, z, a, t) = \min\left(\frac{N_{\text{max,storage}}(i,z,a,t)}{\tau}, \frac{N_{\text{storage}}(i,z,a,t)}{\tau} + \frac{\alpha_{\text{leaf}(i)} C_{\text{leaf}}(i,z,a,t)}{(C{:}N)_{\text{leaf}(i)}} (\alpha_{\text{leaf}})\right) \quad (12)$$

On the last day of each month, plants lose N through seed dispersal ($N_{reproduction}$; kg N day^{-1}) and mortality. Mortality results in the complete transfer of all plant N to the soil.
Seed dispersal is assumed to completely empty the plant's seed bank each month and can be constrained by the amount of both carbon and nitrogen ($C_{storage}$ and $N_{storage}$) in storage.

$$N_{\text{reproduction}}(i, z, a, t) = \min\left(N_{\text{storage}}(i, z, a, t) * f_{\text{seeds}}, \frac{C_{\text{storage}}(i,z,a,t)}{(C{:}N)_{\text{reproduction}(i)}} * f_{\text{seeds}}\right) \quad 13)$$

Here, f_{seeds} is the fraction of stored carbon and nitrogen that will be allocated towards reproduction each month. f_{seeds} = 0.3 for all PFTs.

D. Nitrogen limitation on plant production.

In our model, plants that face N limitation down-regulate their stomatal conductance. A plant's potential N demand (N_{pot}) is defined as what its N demand would have been in the absence of any N limitation. A plant's N supply is given by $N_{avail,soil}$ + BNF_{max}. An **N limitation**

factor (N_{lim})(Supplementary Equation 14), ranging from 0 to 1, where 0 represents severe nitrogen limitation and 1 represents no limitation, is defined as:

$$N_{\text{lim}}(i,z,a,t) = \frac{\frac{N_{\text{avail,soil}}(i,z,a,t)}{\tau} + BNF_{\max}(i,z,a,t)}{N_{\text{pot}}(i,z,a,t) + \frac{N_{\text{avail,soil}}(i,z,a,t)}{\tau} + BNF_{\max}(i,z,a,t)} \quad (14)$$

The model also computes a corresponding water limitation factor (W_{lim}). Stomatal conductance is then down-regulated by the minimum of N_{lim} and W_{lim}.

E. Soil Nitrogen Dynamics

The soil N cycle is similar to that described by ref. 10 for the original ED model. The principal changes are that we now allow for an additional source term (N deposition) and additional sink terms (hydrologic and gas losses) in the **bioavailable N pool (N_{min}; kg N m^{-2})** **(**Supplementary Equation **15).** Nitrogen accumulates in the soil pool each day and receives inputs through N deposition ($N_{deposition}$; 2.47 x 10^{-6} kg N m^{-2} day^{-1}; see next paragraph) and N released through decomposition of plant tissues residing in the soil ($N_{decomposition}$ kg N m^{-2} day^{-1}; see ref. 10). Nitrogen is removed from the bioavailable soil nitrogen pool when plants take up nitrogen for growth or storage ($N_{up,soil}$; kg N m^{-2} day^{-1}) (Supplementary Equation 16) and when nitrogen is lost through leaching ($N_{loss,DIN,leached}$; kg N m^{-2} day^{-1}) (Supplementary Equation 17) or gas emissions($N_{loss,gas}$;kg N m^{-2} day^{-1}) (Supplementary Equation 19).

$$\frac{dN_{\text{min}}(a,t)}{dt} = N_{\text{deposition}}(a,t) + N_{\text{decomposition}}(a,t) - N_{\text{patch,up,soil}}(a,t) - N_{\text{loss,DIN,leached}}(a,t) - N_{\text{loss,gas}}(a,t) \quad (15)$$

The nitrogen taken up from the soil by all trees in a patch **($N_{up,soil}$; kg N m^{-2} day^{-1}) (Supplementary Equation 16)** is the total nitrogen taken up by each plant ($N_{plant,up}$; kg N day^{-1})(Supplementary Equation 6) summed for all plants residing in the same patch.

$$N_{\text{patch,up,soil}}(a,t) = \sum_{i,z} N_{\text{plant,up}}(i,z,a,t) \quad (16)$$

Nitrogen deposition *(N_{dep};* kg N m^{-2} day^{-1}*).* In tropical forests, field measurements and models estimate that 2-10 kg N ha^{-1} year^{-1} are deposited from the atmosphere into soils[13,29-32]. Close to Agua Salud, nitrogen deposition was measured at a rate of 9 kg N ha^{-1} year^{-1} during

2006-2007 (refs. 31-32). We use this observed value, adding nitrogen directly into the bioavailable soil N pool (N_{min}) evenly throughout the year as 2.47 x 10^{-6} kg N m^{-2} day^{-1}.

Hydrologic and gas N losses occur from the bioavailable soil nitrogen pool on a daily time step after plant nitrogen uptake. When soil water content is greater than field capacity, dissolved inorganic nitrogen loss **DIN leaching ($N_{loss,DIN\ leached}$ kg N m^{-2} day^{-1}) (Supplementary Equation 17)** is calculated as a function of the water entering the system after evapotranspiration, the nitrogen concentration in the soil ($N_{conc,soil}$) (Supplementary Equation 18), and a first-order loss factor (E) modifies the amount of nitrogen that can be lost from the bioavailable pool each day.

$$N_{\text{loss,DIN leached}}(a,t) = \left(\text{Precipitation}(a,t) \quad - \text{Evapotranspiration}(a,t)\right) * N_{\text{conc,soil}}(a,t) * E \tag{17}$$

Precipitation (kg water m^{-2} day^{-1}) and evapotranspiration (kg water m^{-2} day^{-1}) are calculated according to hydrology module described in ref. 1 and E = 0.05. A similar loss factor approach is used by the CLM[33] and Biome-BCG models[34]. While the parameterization for E is uncertain, the value was inferred based on the plant carbon accumulation observations over the 300-year chronosequence and is similar to that of other models[27].

Soil nitrogen concentration ($N_{con,soil}$; kg N kg $water^{-1}$) (Supplementary Equation 18) is calculated for the top 1.5 m of the soil[36], reflecting the location of most fine roots in tropical rainforests[35]. $N_{conc,soil}$ depends on the bioavailable nitrogen left in the soil after plant uptake (kg N m^{-2} day^{-1}; numerator of (Supplementary Equation 16)) and the volume of water in the soil profile.

$$N_{\text{conc,soil}}(a,t) = \frac{N_{\text{min}}(a,t) - (N_{\text{patch,up,soil}}(a,t) * \tau)}{\text{Soil}_{\text{water}}(a,t) * \text{Soil}_{\text{depth}} * \rho_{\text{w}}} \tag{18}$$

Here, $\text{Soil}_{\text{water}}$($m^3$ water m^{-3} soil) is the amount of water in the top 9 soil layers and calculated according to hydrology described in ref. 1. ρ_w =1000 kg water m^{-3} water and $\text{Soil}_{\text{depth}}$= 1.5 m.

Using isotopic observations from other tropical rainforests, Houlton et al. (2006) (ref. 36) partitioned the total inorganic nitrogen lost between leaching and gas emissions along a precipitation gradient. Because denitrification measurements are not available at our site, we

apply the relationship developed in Houlton et al. by using the observed precipitation from our tropical forest site at Agua Salud. The partitioning (80% leaching and 20% gas emissions) was then used to estimate the **total inorganic nitrogen lost ($N_{loss, IN}$; kg N m^{-2} day^{-1})** (Supplementary Equation 19) for our tropical forest at Agua Salud:

$$N_{\mathrm{loss,IN}}(a,t) = \frac{N_{\mathrm{loss,DIN\ leached}}(a,t)}{0.8} \qquad (19)$$

and then calculate the **N lost through gas emissions ($N_{loss,gas}$; kg N m^{-2})**

$$N_{\mathrm{loss,gas}}(a,t) = N_{\mathrm{loss,IN}}(a,t) * 0.2 \qquad (20)$$

Using the dissolved organic nitrogen (DON)–precipitation relationship developed for tropical forests from Perakis and Hedin (2002)(ref. 37), we assume a constant rate of 0.0001 kg N m^{-2} yr^{-1} of organic nitrogen($N_{\mathrm{loss,DON,leached}}$) is lost from the decomposition of structural tissues (wood and coarse roots). This loss occurs daily as 2.74 x 10^{-7} kg N m^{-2} day^{-1} as long as structural N is present in the soil. DON never enters the bioavailable nitrogen pool.

We provide our equations that describe plant and soil nitrogen dynamics in a condensed format in Supplementary Tables 2 and 3.

Supplementary Note 1: Evaluation of nitrogen limitation in the individual and ecosystem-level fixation simulations

To determine if the ecosystem and individual level simulations alleviated nitrogen limitation, we compared these simulations with a nitrogen-saturated forest (Supplementary Fig. 1). The nitrogen-saturated forest was initialized with high amounts of soil N, nitrogen losses were turned off from the system, and extra N fertilizer was added to the forest throughout succession. We found a slight enhancement of forest carbon in the nitrogen-saturated forest compared to the individual level fixation simulation, especially in the first 100 years, indicating that the forest in the individual level simulation still experienced nitrogen limitation. We found no difference between the ecosystem level simulation and the nitrogen saturated forest indicating that the ecosystem level simulation did not experience nitrogen limitation.

We did find, however, that the forest simulations with individual-level fixation were still nitrogen limited. Why is there still a small amount of nitrogen limitation in the individual level fixation forest when fixers are present? First, the maximum fixation rate parameterized in our model internalized other nutrient, physiological, or energy constraints on how much nitrogen could be acquired through N_2 fixation. Second, there may be lag time in the redistribution of nitrogen from fixers to non-fixers, which would keep non-fixers nitrogen limited in the non-fertilized forest. Third, competitive interactions and costs of fixation may have prevented fixers from being more abundant and fixing more nitrogen in the forest to completely alleviate nitrogen limitation.

Supplementary Note 2: Scaling up the fixation effect to Neotropical forests

We scaled up our fixation effect to evaluate the consequences for carbon storage across two types of tropical forests: (1) forest areas that have been cleared and that have been pledged for reforestation under the Bonn Challenge, and (2) mature tropical forests that currently exist. The estimates were constrained to Neotropical forests, since the species identities and abundance of nitrogen-fixing trees are similar to our field study.

First, we combined analyses to estimate the effects of fixation on tropical reforestation across Neotropical areas pledged under the Bonn Challenge[38]. In December 2016, we downloaded the area of land committed for reforestation by each Neotropical country from the Bonn Challenge website[38]: Brazil, Columbia, Costa Rica, Ecuador, El Salvador, Guatemala, Honduras, Mexico (including Quintana Roo and Campeche – Yucatan), Nicaragua, Panama and Peru. We applied a mean carbon sequestration rate observed across Neotropical secondary forests (122 Mg ha^{-1} over the first 20 years; from ref. 39) to all forest areas. We then applied our estimate that forests with fixers store 85% more carbon in the first few decades of recovery than forests without fixers by dividing by 1.85. The difference between these estimates was the additional carbon stored due to fixation. We converted carbon to CO_2 using the mass ratio (3.67).

Second, we scaled up our finding that fixation increases carbon storage in mature forests by 10% to estimate how much carbon this would account for in Neotropical forests where fixer densities and identity are similar to our study sites[40]. (1) We determined the quantity of carbon stored in Neotropical forests by multiplying the area of the Neotropics that contains intact tropical forest (773.2 M ha; Supplementary Information Table 2 in ref. 41) by the carbon density

for tropical rainforests (145 Mg C ha^{-1}; Table 1 in ref. 42) (112 Pg C). (2) We calculated the carbon stored in a forest without fixation by dividing the Neotropical carbon value by 1.1 (101.9 Pg C). (3) We found the additional carbon stored in Neotropical forests due to fixation by multiplying this quantity by 0.1 (10.1 Pg C).

Supplementary Note 3: Sensitivity analysis of model to parameter values for the nitrogen fixer PFT and disturbance

We carried out a parameter sensitivity analysis to investigate how parameter uncertainty affected the simulations of observed variables. The observed variables selected for analysis included the time series of plant biomass, basal area, and N_2 fixed (cf. Figs. 1a, 1b, and 2a in the main text). Because parameter sensitivity of ED2 has already been carried out for tropical forests[3-4] as well as in other contexts[5,43-44], we here focused only on a subset of model parameters of particular relevance to our study. This subset included the (i) maximum specific rate of N_2 fixation, (ii) the maximum carboxylation rate of the nitrogen-fixing PFT, (iii) the rate of canopy gap formation created by treefall disturbance, (iv) the soil nitrogen leaching loss factor, and (v) the maximum amount of nitrogen that can be stored by plant cohorts.

First, the maximum specific rate of N_2 fixation imposed an important constraint on our ecosystem simulations. Because observed rates were variable[11], we analyzed parameter values that completely encompassed the observed values by considering our simulation without fixation which prescribes a zero rate of N_2 fixation, as well as non-zero rates that ranged from 1×10^{-6} to 7×10^{-4} (kg N fixed) (kg tree dry biomass)$^{-1}$ (day)$^{-1}$. Second, previous studies[5] have identified the maximum carboxylation rate as an important model parameter. Given that multiple nitrogen-fixing species were present at our study site, and that species abundance was sensitive to stand age, we explored a wide range of values, from 10 to 54 μmol m^{-2} s^{-1}. Third, we varied the rate of canopy gap formation to capture the observed variability of disturbance in neotropical forests[45] and because this parameter represents a defining feature of ED2[10]. Specifically, we prescribed rates ranging from 0.002 to 0.02 $year^{-1}$. Fourth, because our model of soil nitrogen dynamics does not explicitly distinguish between soluble and insoluble inorganic N, we investigate the sensitivity to the prescribed loss factor (E). We allow this factor to range from 0 to 0.3. For comparison, the CLM model[33] uses a value of 0.1. Fifth, the maximum nitrogen storage capacity

of each cohort, measured in units of the amount of nitrogen in a full canopy of leaves, was allowed to range from 0.33 to 5.

Sensitivity analysis was conducted one parameter at a time. When the specific rate of N_2 fixation was varied, all other parameters subject to sensitivity analysis were held at their baseline values. For parameters other than the specific rate of N_2 fixation, sensitivity analysis was carried out under both the baseline N_2 fixation rate and zero N_2 fixation.

The modeled biomass (Supplementary Fig. 4a), basal area (Supplementary Fig. 4b), and N_2 fixed (Supplementary Fig. 4c) exhibited step-like sensitivity to the maximum specific fixation rate. For maximum specific fixation rates below about 3 x 10^{-5} (kg N fixed) (kg tree dry biomass)$^{-1}$ (day)$^{-1}$, the ecosystem variables behaved similarly to the case of zero-fixation. As the maximum specific fixation rate increased beyond 3 x 10^{-5} (kg N fixed) (kg tree dry biomass)$^{-1}$ (day)$^{-1}$, the ecosystem variables rapidly increased but then showed little sensitivity to further increases in the maximum specific fixation rate. Thus, the strong effect of fixation reported in this paper is relatively insensitive to the maximum fixation rate parameter outside of the very low end of our observed maximum fixation rates.

Biomass and basal area were sensitive to the N_2 fixer maximum carboxylation rate (Supplementary Fig. 5a-b). Generally, larger rates led to more biomass and basal area accumulation. Smaller rates led to the fixer PFT fixing less carbon, and hence having less need for N_2 fixation to maintain stoichiometry (Supplementary Fig. 5c). The effect of fixation on biomass (biomass from simulations with baseline N_2 fixation rate minus biomass from simulations with zero N_2 fixation) also depended on the maximum carboxylation rate (Supplementary Fig. 6). While all time series were qualitatively similar, the magnitude of the effect was strongest for the largest maximum carboxylation rate. Large carboxylation rates led to large nitrogen demand, and thus amplified the significance of the fixation trait.

Larger gap creation rates led to reduced biomass (Supplementary Fig. 7a) and basal area (Supplementary Fig. 7b). In the latter half of the simulations, a larger gap creation rate led to increased N_2 fixation (Supplementary Fig. 7c). This correlation existed because a larger gap creation rate generally led to more light availability, and increased light availability tended to favor PFTs with a large nitrogen demand. Furthermore, more light availability would provide the energy needed to pay for costly fixation. We found that a larger gap creation rate increased the biomass difference between simulations with baseline N_2 fixation to those with zero N_2 fixation

(Supplementary Fig. 8). Again, this can be understood in terms of light availability: the more light, the larger the nitrogen demand, and thus the greater the importance of fixation as a means for meeting that demand.

Biomass (Supplementary Fig. 9a) and basal area (Supplementary Fig. 9b) were modestly sensitive to the inorganic N loss factor. Larger values of this factor led to increased rates of N loss, and thus somewhat smaller biomass and basal area. In compensation for increased losses at larger values of the loss factor, increases in the loss factor were associated with increases in the N2 fixation rate (Supplementary Fig. 9c). These differences were most evident in the latter part of our simulated time series. Correspondingly, larger values of the loss factor increased the biomass difference between simulations with baseline N_2 fixation to those with zero N_2 fixation (Supplementary Fig. 10).

Under our baseline N_2 fixation rate, variation in the maximum nitrogen storage capacity had little effect on either biomass, basal area, or total N_2 fixation (Fig. 11). These results suggest that N_2 fixation may be able to buffer against variation in nitrogen storage capacity. Indeed, simulations without N_2 fixation exhibited somewhat stronger sensitivity of biomass to nitrogen storage capacity: the spread in the biomass difference plot (Fig. 12) is larger than the spread in the simulations with baseline N_2 fixation. Nevertheless, all curves in the biomass plot (Fig. 12) are qualitatively similar.

Collectively, these results lead to four conclusions. First, the model shows little sensitivity of key observables (biomass accumulation, basal area, and N_2 fixed) to the specific maximum rate of N_2 fixation. The caveat is that the maximum fixation rate must be above a certain threshold for this result to hold; importantly, this situation is indeed supported by field observations. Second, biomass differences between the baseline-fixation simulation and the zero-fixation simulation are qualitatively similar regardless of the value of either the maximum carboxylation rate, the gap creation rate, the inorganic N loss factor, or the maximum N storage capacity. Third, the maximum carboxylation rate, the gap creation rate, and (to a lesser extent) the inorganic N loss factor do affect our simulations. As these parameters increase, the effect of fixation on biomass becomes amplified. Fourth, N_2 fixation is able to buffer against variation in the maximum nitrogen storage capacity factor.

Supplementary Note 4: Calculation in support of Fig. 3d

We carried out the following calculation to generate the observed data on per-hectare basal area across three functional plant types and three successional ages. The samples were collected in 0.1 ha plots for forests aged 5 and 30 years, and 1 ha plots for forests aged 300 years as described in the main text.

We calculated the relative abundance of the different plant functional types based on the following steps (see tables below): Step 1: We selected the 5 most abundant species by basal area in each forest age (5, 30 and 300 years) across all plots. Step 2: We calculated the total basal area (per ha) of each dominant species in each forest age (summed across all plots for a given age) and the total basal area across all forest ages. Step 3: We calculated the relative abundance of each species in each forest age as the percent fraction of the total basal area of that species across all three forest ages. Step 4: Finally, we calculated the mean relative basal area for each plant functional type (PFT) as the mean across the 5 most abundant species at each forest age.

Step 1 - Most abundant species in each forest age

FOREST AGE	Dominant species across all plots
5	BYRSCR
5	CONOXA
5	VISMBA
5	VISMMA
5	MICOAR
30	MICOPO
30	BROSCO
30	POULAR
30, 300	POURBI
30, 300	TAPIGU
300	TERMAM
300	WELFRE
300	TAB1GU

STEP 2 - Total basal area per species (BA / 4 ha)

PFT/Species	BA age 5	BA age 30	BA age 300	Total ba across 3 ages
BYRSCR	3.0	0.1	0.0	3.0
CONOXA	5.3	0.0	0.0	5.3
VISMBA	2.3	0.2	0.0	2.4
VISMMA	2.0	0.2	0.0	2.2
MICOAR	1.0	0.1	0.0	1.1
MICOPO	0.0	7.7	0.0	7.7
BROSCO	0.0	5.3	1.3	6.6
POULAR	0.0	11.6	1.1	12.7
POURBI	0.0	4.1	5.4	9.5
TAPIGU	0.0	3.6	18.0	21.6
TERMAM	0.4	0.0	5.4	5.9
WELFRE	0.0	0.0	5.7	5.7
TAB1GU	0.0	0.0	5.4	5.4
Fixer	1.4	6.7	7.6	15.7

STEP 3 - Relative basal area per species (% BA)

PFT/Species	relative BA age 5	relative BA age 30	relative BA age 300
BYRSCR	98.3	1.7	0.0
CONOXA	100.0	0.0	0.0
VISMBA	93.4	6.5	0.1
VISMMA	93.1	6.9	0.0
MICOAR	92.5	5.9	1.5
MICOPO	0.0	100.0	0.0
BROSCO	0.0	80.4	19.6
POULAR	0.0	91.2	8.8
POURBI	0.0	43.4	56.6
TAPIGU	0.0	16.7	83.3
TERMAM	6.9	0.0	93.1
WELFRE	0.0	0.0	100.0
TAB1GU	0.0	0.0	100.0
Fixer	8.9	42.6	48.5

**STEP 4 - Basal area (% BA) by functional type averaged across 5 species at each forest age**					
PFT/Forest age specialist	mean relative BA age 5	mean relative BA age 30	mean relative BA age 300	number of plots	total area summed across
year 5 specialist	95.5	4.2	0.3	10	2.8
year 30 specialist	0.0	66.3	33.6	10	2.8
year 300 specialist	1.4	12.0	86.6	10	2.8
Fixer	8.9	42.6	48.5	10	2.8

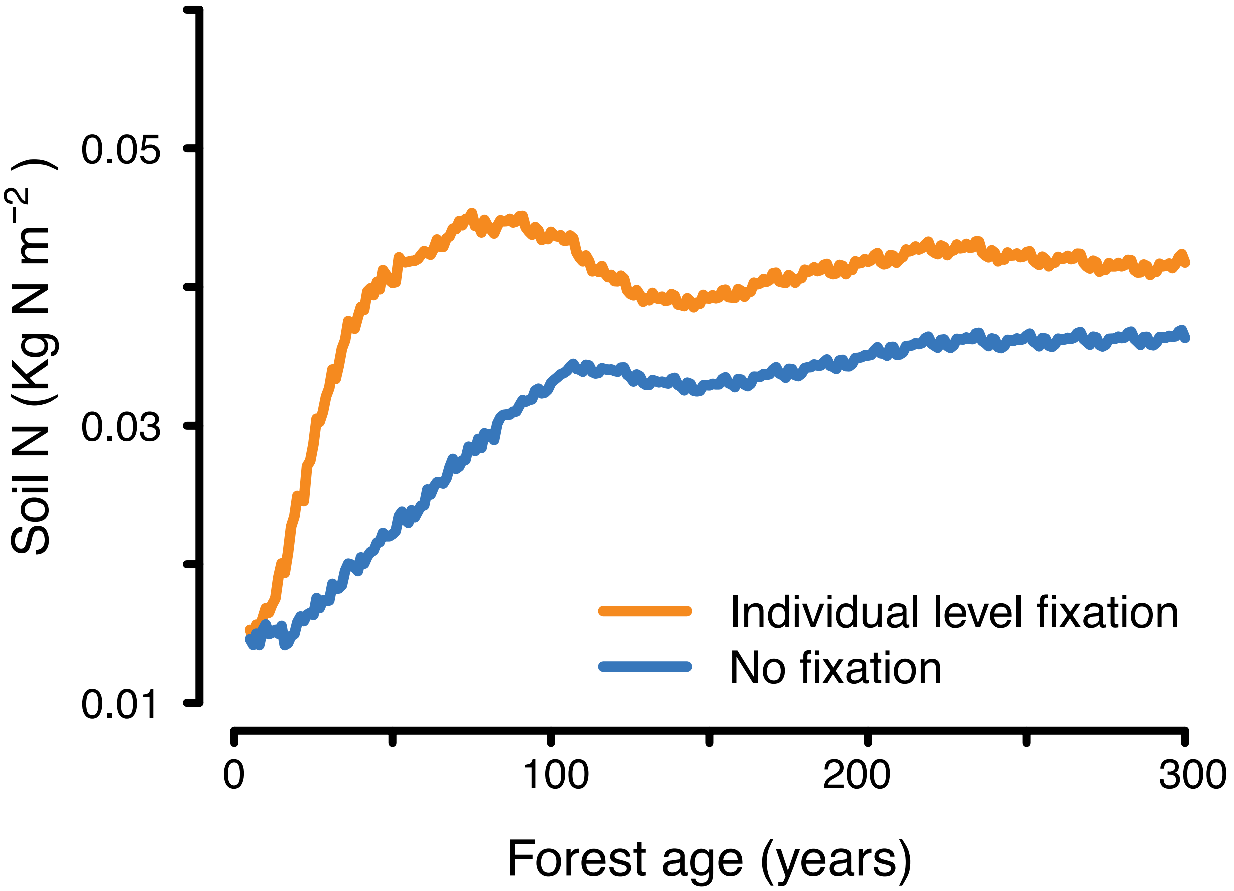

Supplementary Figure 1. Comparison of soil N in simulations with and without nitrogen fixation used to calculate the direct and indirect effects of nitrogen fixation (Figure 3c). Soil N increases at a faster rate in early succession in simulations that have fixation than in simulations without fixation.

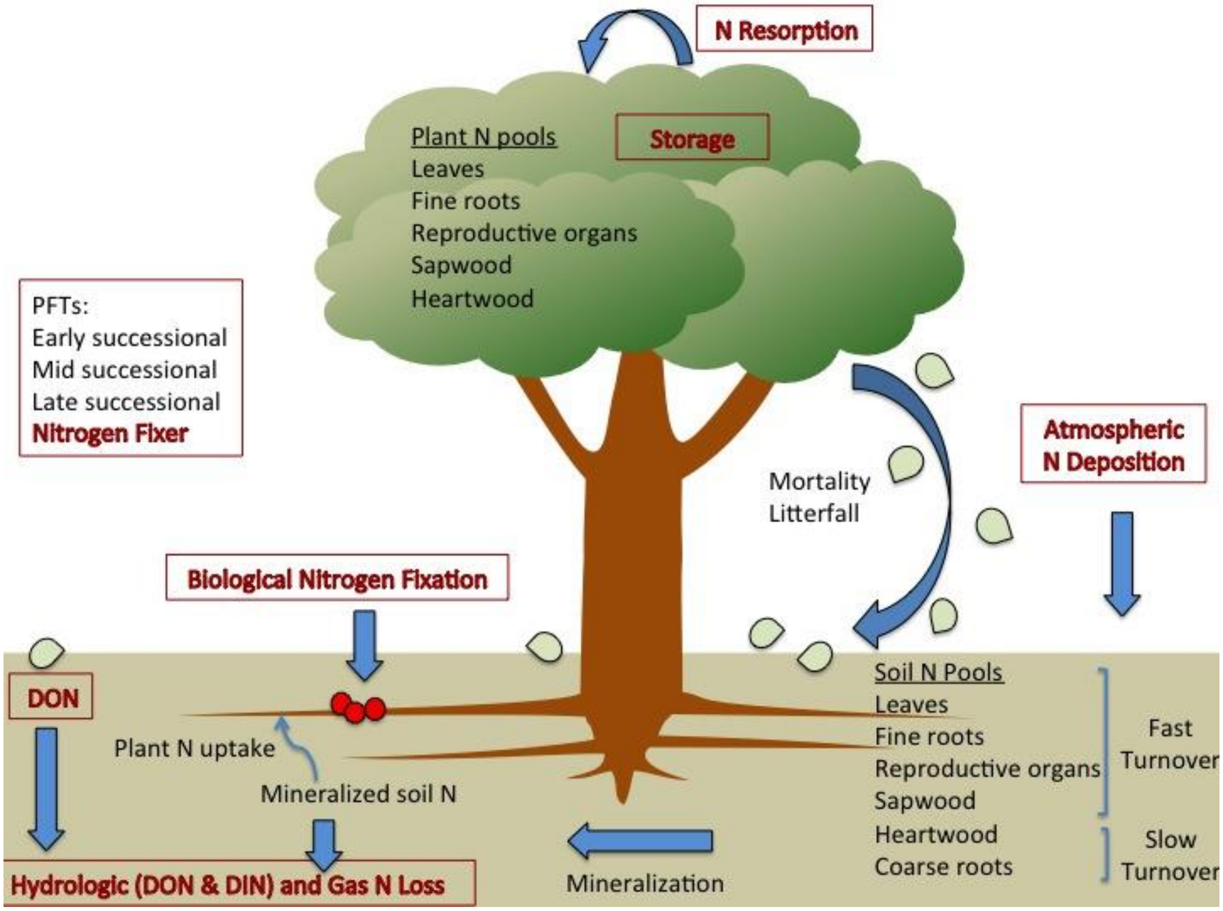

Supplementary Figure 2. Diagram of the nitrogen cycle in ED2. Bold red text displays new additions to the cycle.

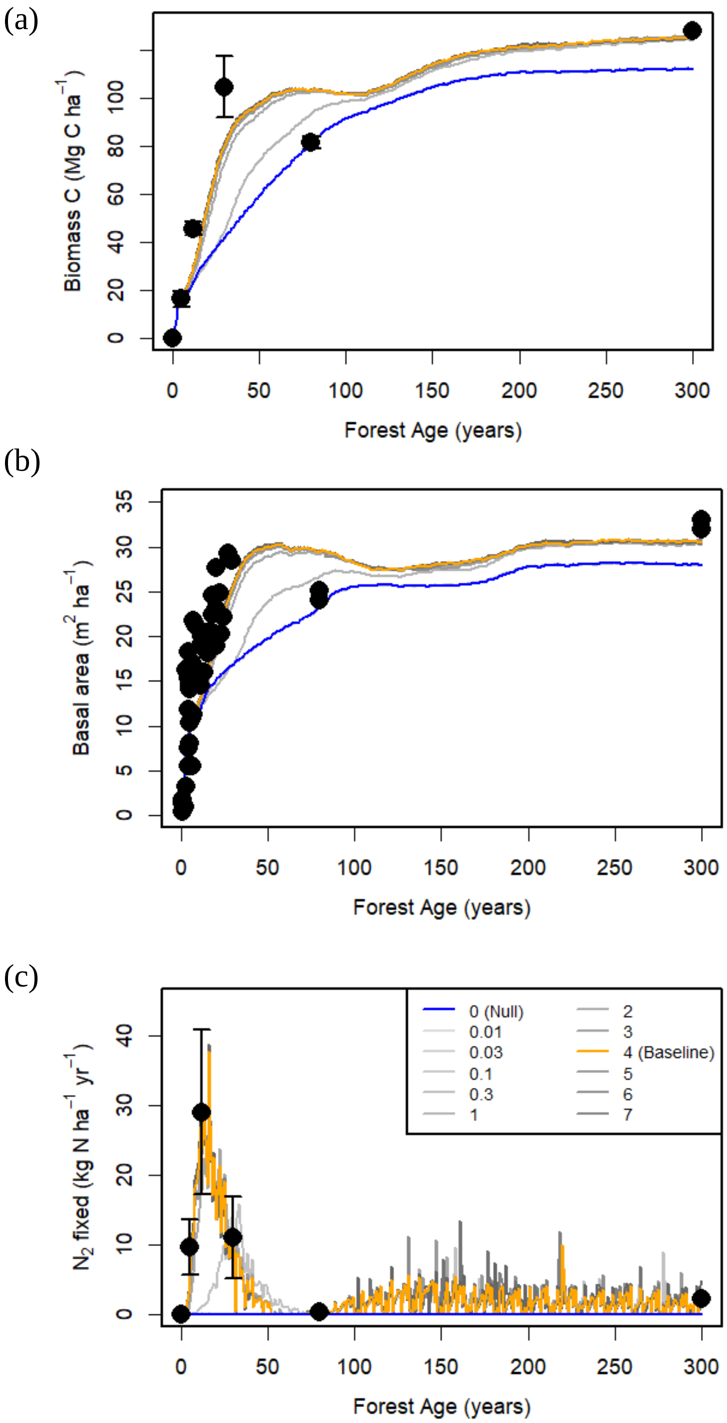

Supplementary Figure 3. Sensitivity of plant biomass (a), basal area (b), and N_2 fixed (c) to the maximum specific N_2 fixation rate. The color code is given in panel (c) and the units are 10^{-4} (kg N fixed) (kg plant biomass)$^{-1}$ (day)$^{-1}$. The observations are given as points.

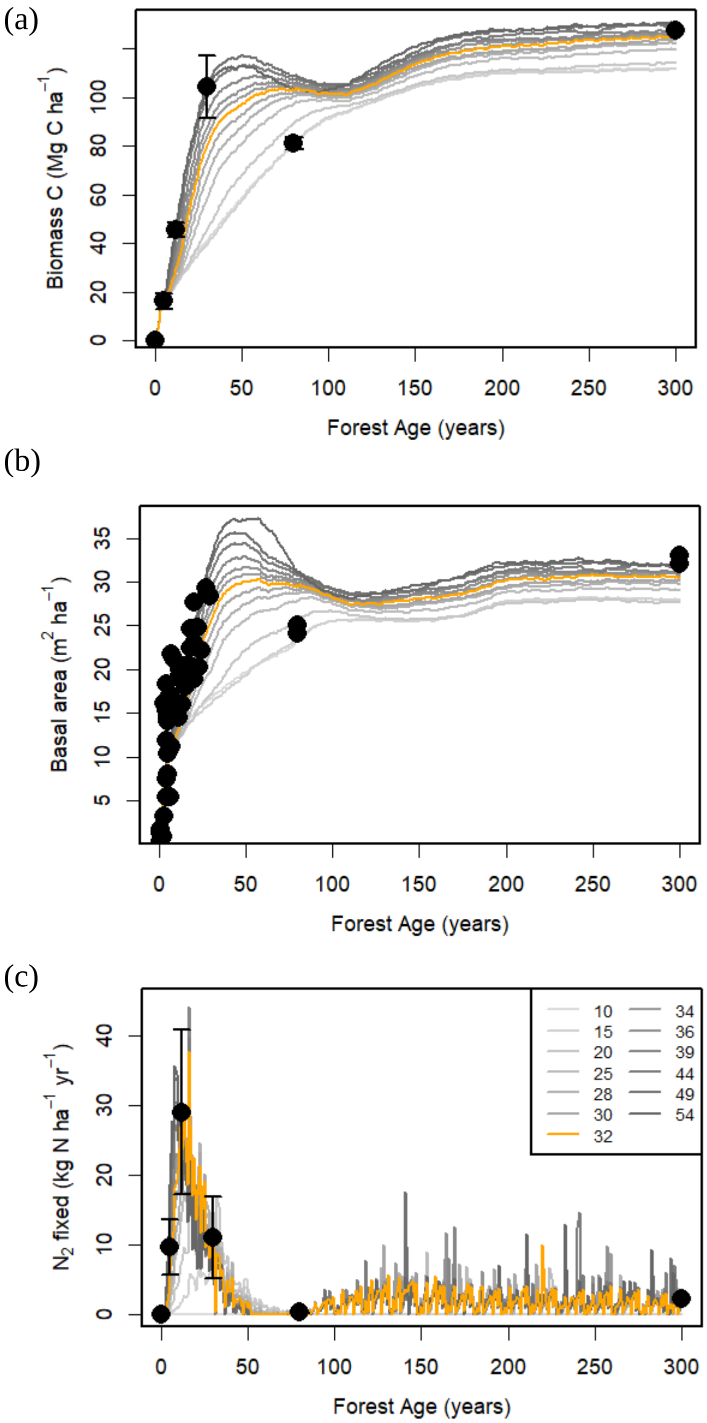

Supplementary Figure 4. Sensitivity of plant biomass (a), basal area (b), and N_2 fixed (c) to the maximum carboxylation rate of the N-fixer PFT. The color code is given in panel (c) and the units are μmol m^{-2} s^{-1}. The observations are given as points.

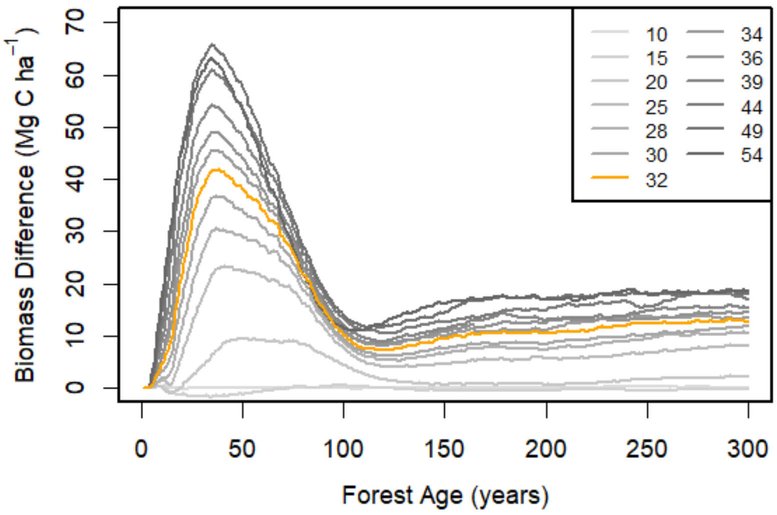

Supplementary Figure 5. Effect of N_2 fixation on biomass for different values of the maximum carboxylation rate. The sign convention is "simulation with baseline fixation" minus "simulation with zero fixation". The color code for the maximum carboxylation rate is given in the legend, and the units are μmol m^{-2} s^{-1}. The orange line corresponds to the baseline maximum carboxylation rate.

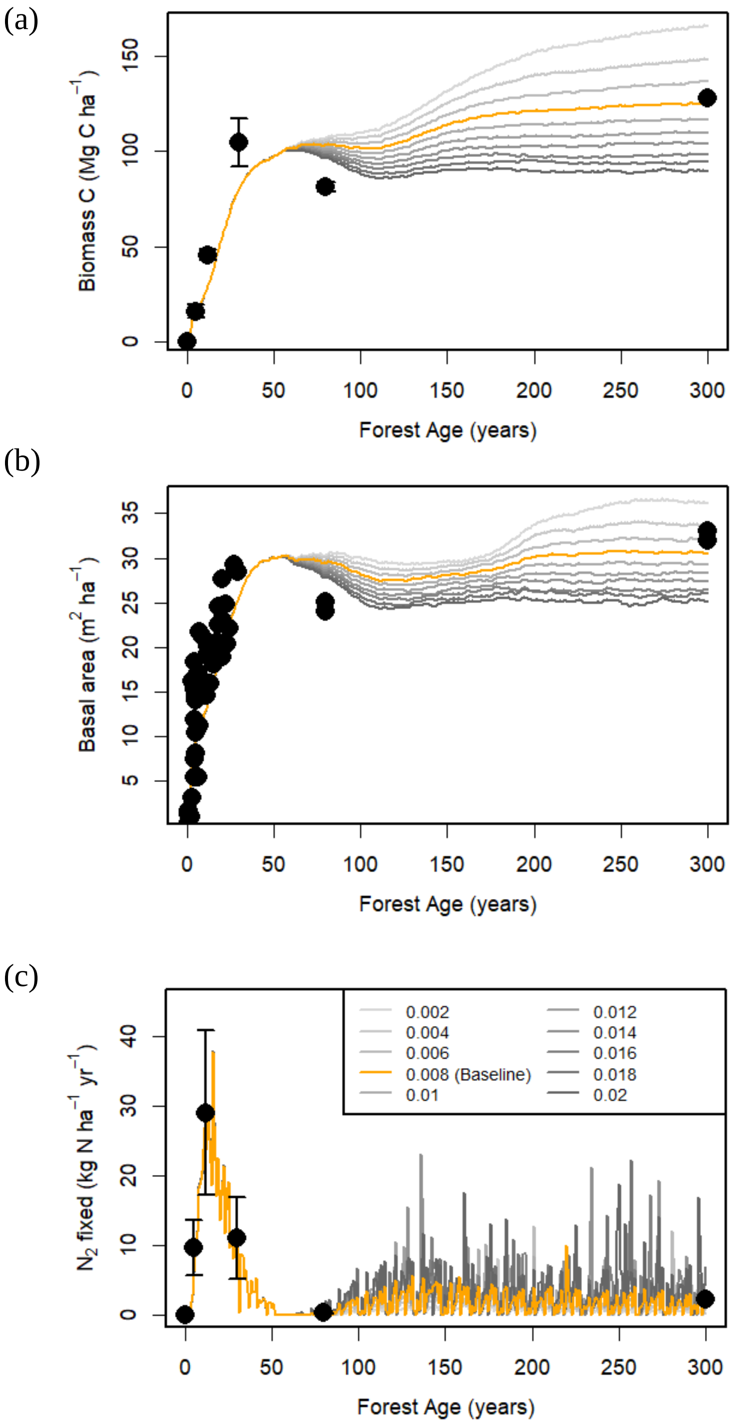

Supplementary Figure 6. Sensitivity of simulated plant biomass (a), basal area (b), and N_2 fixed (c) to the canopy gap creation rate. The color code is given in panel (c) and the units are year^{-1}. The observations are given as points.

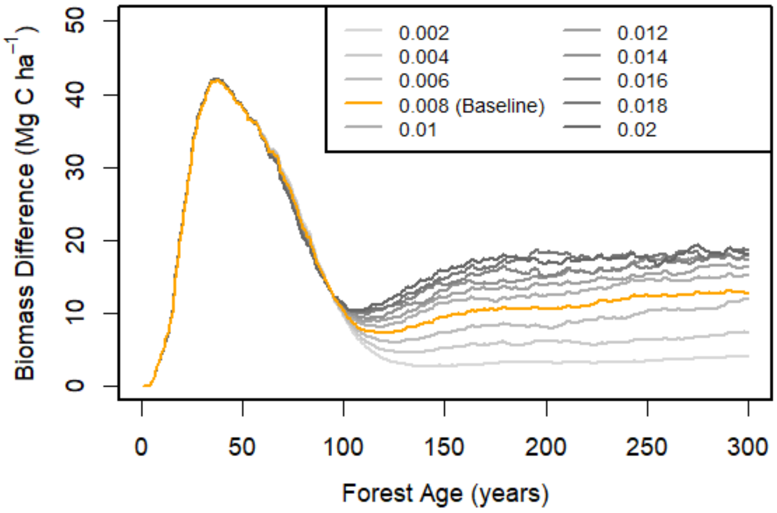

Supplementary Figure 7. Effect of N_2 fixation on biomass for different values of the gap creation rate. The sign convention is “simulation with baseline fixation” minus “simulation with zero fixation”. The color code for the gap creation rate is given in the legend, and the units are year^{-1}. The orange line corresponds to the baseline gap creation rate.

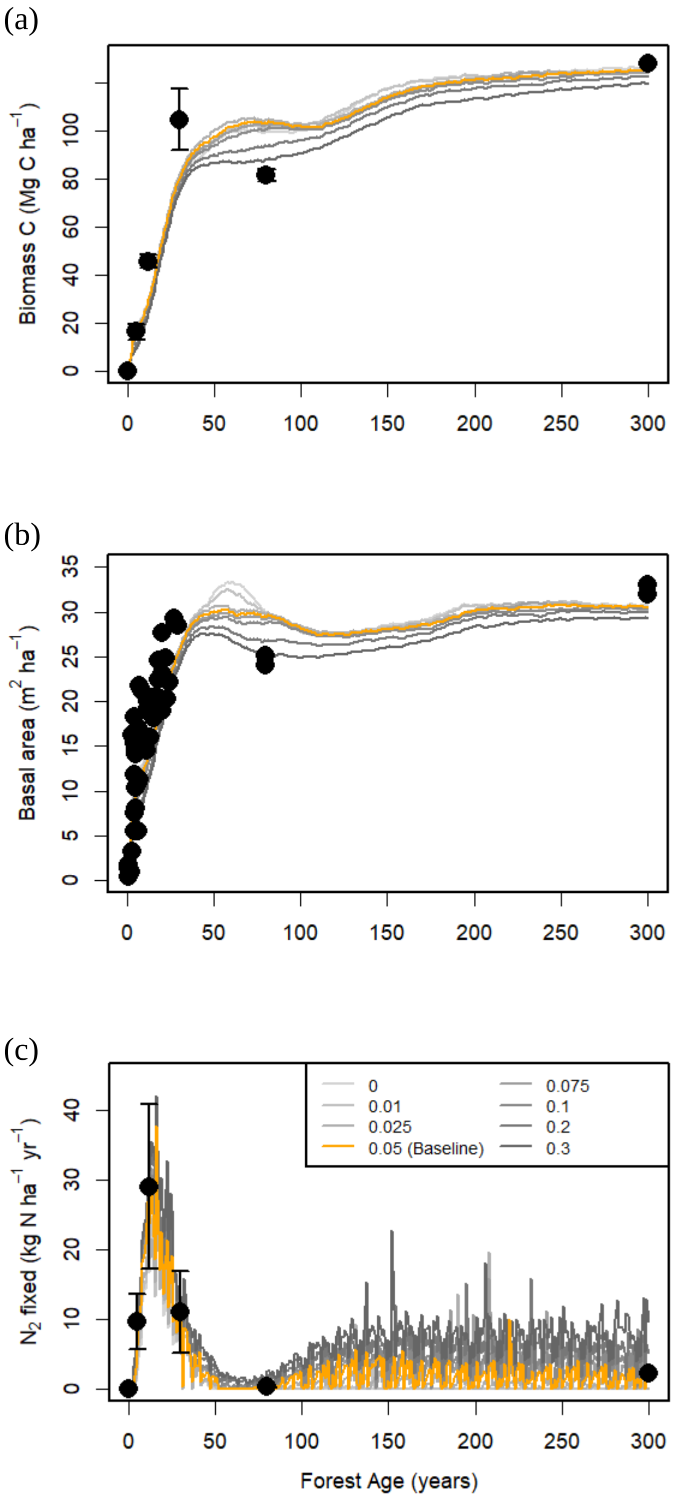

Supplementary Figure 8. Sensitivity of simulated plant biomass (a), basal area (b), and N_2 fixed (c) to the inorganic N loss factor. The color code is given in panel (c). The observations are given as points.

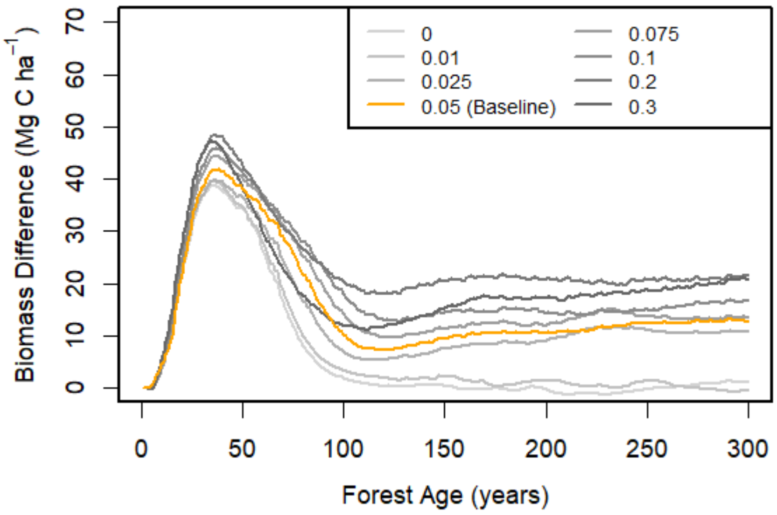

Supplementary Figure 9. Effect of N_2 fixation on biomass for different values of the inorganic N loss factor. The sign convention is “simulation with baseline fixation” minus “simulation with zero fixation”. The color code for the parameter is given in the legend. The orange line corresponds to the baseline parameter value.

(a)

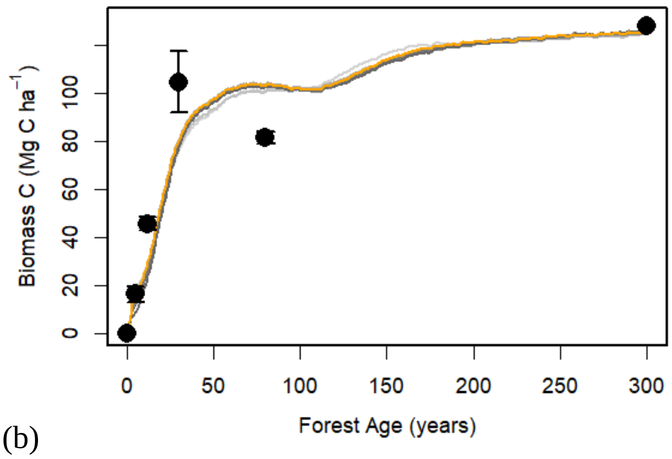

(b)

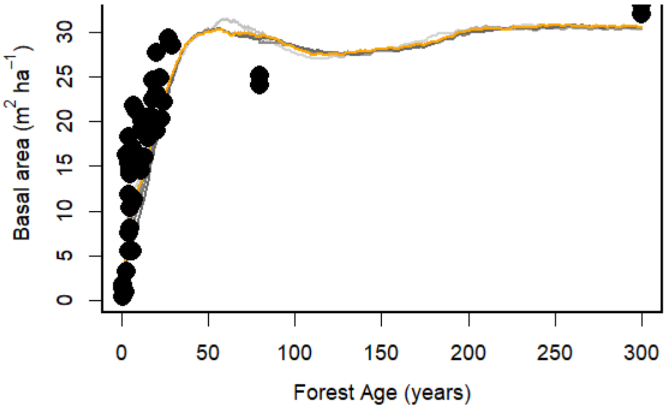

(c)

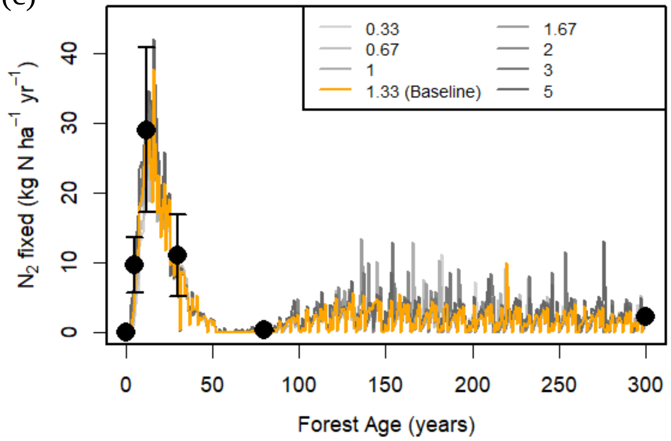

Supplementary Figure 10. Sensitivity of simulated plant biomass (a), basal area (b), and N_2 fixed (c) to the maximum N storage capacity. The color code is given in panel (c). The observations are given as points.

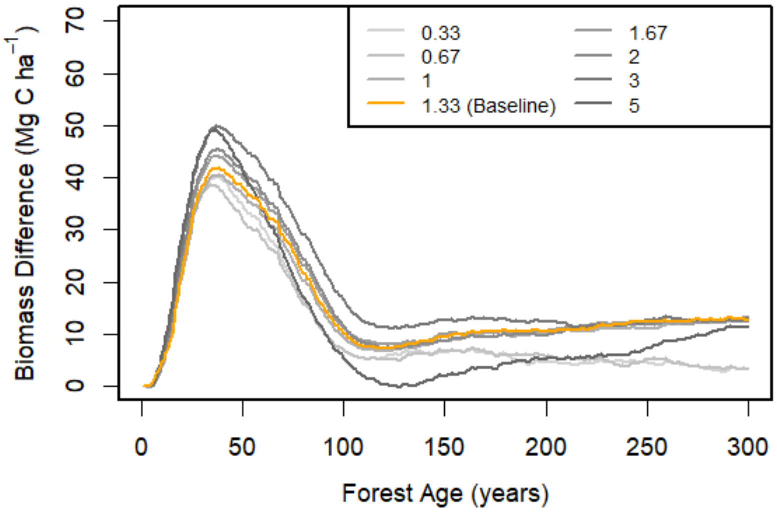

Supplementary Figure 11. Effect of N_2 fixation on biomass for different values of the maximum N storage capacity. The sign convention is "simulation with baseline fixation" minus "simulation with zero fixation". The color code for the parameter is given in the legend. The orange line corresponds to the baseline parameter value.

Supplementary Table 1. Model parameter values and definitions for the nitrogen cycle in ED2. *indicates that variables are based on calculations described in ref. 1 and parameter values in ref. 5.

Parameter	Description	Equation the parameter is defined in (Equations the parameter is used in)	Value
α_{leaf}	Leaf turnover rate	*(11,12)	*
α_{root}	Fine root turnover rate	*(11)	*
$^{\alpha}{}_{leaf}$	Retranslocation fraction before leaf shedding	constant(12)	0.48
α	Gap age	*	
Biomass	Above and belowground biomass	*(8)	
BNF	Biological Nitrogen Fixation	9(10)	
BNF_{max}	Maximum daily BNF	8(9,14)	
BNF_{rate}	Rate of BNF	Constant (8)	0.00039
BNF_{want}	N wanted through BNF	7(9)	
BNF_{cost}	Carbon cost of BNF	Constant (7)	9.12
C_{crown}	C stored in a full crown. Calculated from dbh and plant allometry for the PFT	*(3)	
C_{leaf}	C stored in leaves	*(11,12)	
C_{root}	C stored in fine roots	*(11)	
$C_{storage}$	C in storage reservoir	*(13)	
$(C:N)_j$	C:N of the plant structure that carbon will be allocated to during growth	*(1,7)	
$(C:N)_{leaf}$	C:N of the leaf	*(3,11,12)	
$(C:N)_{root}$	C:N of the fine roots	*(5,11)	
$(C:N)_{reproduction}$	C:N of the reproductive structures	*13)	
dC_j/dt	Carbon available for growth	*(1)	
E	Efficiency factor	Constant (17)	0.05
Evapotranspiration		*(17)	
f_{seeds}	Fraction of stored nutrents that can be used towards reproduction	*(13)	0.3
i	PFT type	Early, mids,lates,fixers	*
j	Plant tissue (leaves, fine roots, wood, reproductive structures)	*(I,7)	
$N_{avail,soil}$	Soil N available to the plant	5(4,6,7,14)	
$N_{conc,soil}$	Concentration of mineralized N in soil	18(17)	

Supplementary Table 1. (continued)

Parameter	Description	Equation the parameter is defined in (Equations the parameter is used in)	Value
$N_{daily,plant,loss}$	Plant N loss	11	
$N_{decomposition}$	Nitrogen released into bioavailable pool from decomposition	*(15)	
$N_{deposition}$	Nitrogen Deposition	Constant(15)	2.47 x 10-6
N_{lim}	Nitrogen limitation factor	14	
$N_{loss,DIN,leached}$	Dissolved inorganic N lost from mineralized N pool through leaching	17(15,19)	
$N_{loss, IN}$	Total inorganic N loss	19(20)	
$N_{loss,gas}$	Inorganic N lost from mineralized N pool through gas emissions	20(15)	
N_{min}	Mineralized N available to plants (bioavailable N)	change over time described in 15, (5,18)	
$N_{max,storage}$	Maximum N in storage	3(2,12)	
$N_{patch,up,soil}$	Plant nitrogen uptake from soil	16(15,18)	
$N_{plant,up}$	Plant N uptake from soil and BNF	10(16)	
$N_{plant,up,soil}$	Plant N uptake from soil	6(10)	
N_{pot}	N wanted for growth in the absence of any N limitation	*(14)	
$N_{reproduction}$	N in biomass of new saplings	13 (4)	
$N_{resorption}$	Nitrogen resorbed before leaf shedding	12(4)	
$N_{storage}$	N in storage	4(2,12,13)	
$N_{want, growth}$	N wanted for growth	1(4,6,7)	
$N_{want, storage}$	N wanted for storage	2(4,6)	
Precipitation		*(17)	
ρ_w	Water density	constant(18)	1000
$S_{closure}$	Stomatal conductance limitation	Described in text (S Table 2)	
$Soil_{depth}$	Depth of soil profile where N is considered for Nconc,soil	Constant (18)	1.5
$Soil_{water}$	Water in the top 9 soil layers	*(18)	
t	time		*
W_{lim}	Water limitation factor	*(S Table 2)	
z	plant size	*	
T	The daily time step	Constant	1

Supplementary Table 2. Model equations for the plant nitrogen dynamics in ED2. See supplementary Table 1 for parameter definitions, values, and units.

Eqn no.	Function	Equation	Units
1	N wanted for growth	$N_{\text{demand,growth}}(i,z,a,t) = \sum_j \frac{1}{(C:N)_j(i)}\left(\frac{dC_j(i,z,a,t)}{dt}\right)$	kg N day-1
2	N wanted for storage	$N_{\text{want,storage}}(i,z,a,t) = \frac{N_{\text{max,storage}}(i,z,a,t) - N_{\text{storage}}(i,z,a,t)}{\tau}$	kg N day-1
3	Maximum N in storage	$N_{\text{max,storage}}(i,z,a,t) = 1.33 \frac{C_{\text{crown}}(i,z,a,t)}{(C:N)_{\text{leaf}}(i)}$	kg N
4	Change in N_{storage}	$\frac{dN_{\text{storage}}(i,z,a,t)}{dt} = N_{\text{resorption}}(i,z,a,t) - N_{\text{reproduction}}(i,z,a,t) +$ $\min(N_{\text{want,storage}}(i,z,a,t), (\frac{N_{\text{avail,soil}}(i,z,a,t)}{\tau}, N_{\text{demand,growth}}(i,z,a,t))$	kg N day-1
5	Soil N available	$N_{\text{avail,soil}}(i,z,a,t) = \frac{C_{\text{root}}(i,z,a,t)}{\sum_{i,z} C_{\text{root}}(i,z,a,t)} * N_{\text{min}}(a,t)$	kg N
6	N uptake from soil for an individual plant	$N_{\text{plant,up,soil}}(i,z,a,t)$ $= \min(N_{\text{demand,growth}}(i,z,a,t) + N_{\text{want,storage}}(i,z,a,t), \frac{N_{\text{avail,soil}}(i,z,a,t)}{\tau})$	kg N day-1
7	N wanted through biological N_2 fixation	$BNF_{\text{want}}(i,z,a,t) = \frac{(N_{\text{demand,growth}}(i,z,a,t) - \frac{N_{\text{avail,soil}}(i,z,a,t)}{\tau}) * (C:N)_{\text{leaf}}(i)}{BNF_{\text{cost}} + (C:N)_{\text{leaf}}(i)}$	kg N day-1
8	Maximum biological N_2 fixation	$\text{BNF}_{\text{max}}(i,z,a,t) = \text{Biomass}(i,z,a,t) * \text{BNF}_{\text{rate}}$	unitless
9	Actual biological N_2 fixation	$\text{BNF}(i,z,a,t) = \min(\text{BNF}_{\text{want}}(i,z,a,t), \text{BNF}_{\text{max}}(i,z,a,t))$	unitless

Supplementary Table 2(continued). Model equations for the plant nitrogen dynamics in ED2. See supplementary Table 1 for parameter definitions, values, and units.

Eqn no.	Function	Equation	Units
10	Total plant N uptake from soil and BNF	$N_{\text{plant,up}}(i,z,a,t) = N_{\text{plant,up,soil}}(i,z,a,t) + \text{BNF}(i,z,a,t)$	kg N day-1
11	Plant N loss from leaf and root turnover	$N_{\text{daily,plant,loss}}(i,z,a,t) = \frac{\alpha_{\text{leaf}}(i)C_{\text{leaf}}(i,z,a,t)}{(C:N)_{\text{leaf}}(i)} + \frac{\alpha_{\text{root}}(i)C_{\text{root}}(i,z,a,t)}{(C:N)_{\text{root}}(i)}$	kg N day-1
12	N resorbed before leaf shedding	$N_{\text{resorption}}(i,z,a,t) = \min\left(\frac{N_{\text{max,storage}}(i,z,a,t)}{\tau}, \frac{N_{\text{storage}}(i,z,a,t)}{\tau} + \frac{\alpha_{\text{leaf}}(i)C_{\text{leaf}}(i,z,a,t)}{(C:N)_{\text{leaf}}(i)}\alpha_{\text{leaf}}(i)\right)$	kg N day-1
13	N taken from storage for reproduction	$N_{\text{reproduction}}(i,z,a,t) = \min\left(N_{\text{storage}}(i,z,a,t) * f_{\text{seeds}}, \frac{C_{\text{storage}}(i,z,a,t)}{(C:N)_{\text{reproduction}}(i)} * f_{\text{seeds}}\right)$	kg N day-1
14	Nitrogen limitation factor	$N_{\text{lim}}(i,z,a,t) = \frac{\frac{N_{\text{avail,soil}}(i,z,a,t)}{\tau} + \text{BNF}_{\text{max}}(i,z,a,t)}{N_{\text{pot}}(i,z,a,t) + \frac{N_{\text{avail,soil}}(i,z,a,t)}{\tau} + \text{BNF}_{\text{max}}(i,z,a,t)}$	unitless
Described in text	Stomatal conductance limitation	$S_{\text{closure}}(i,z,a,t) = \min(N_{\text{lim}}(i,z,a,t), W_{\text{lim}}(i,z,a,t))$	unitless

Supplementary Table 3. Model equations for soil nitrogen dynamics in ED2. See Supplementary Table 1 for parameter definitions, values, and units.

Eqn no.	Function	Equation	Units
15	Change in mineralized soil N pool	$\frac{dN_{\text{min}}(a,t)}{dt} = N_{\text{deposition}}(a,t) + N_{\text{decomposition}}(a,t) - N_{\text{patch,up,soil}}(a,t) - N_{loss\text{,DIN,leached}}(a,t) - N_{\text{loss,gas}}(a,t)$	kg N m^{-2} day^{-1}
16	N uptake from soil for all plants in a patch	$N_{\text{patch,up,soil}}(a,t) = \sum_{i,z} N_{\text{plant,up}}(i,z,a,t)$	kg N m^{-2} day^{-1}
17	Dissolved inorganic N lost from mineralized N pool	$N_{\text{loss,DIN leached}}(a,t) = (\text{Precipitation} - \text{Evapotranspiration}(a,t)) * N_{\text{conc,soil}}(a,t) * E$	kg N m^{-2} day^{-1}
18	Concentration of bioavailable N in the soil	$N_{\text{conc,soil}}(a,t) = \frac{N(a,t) - (N_{\text{patch,up,soil}}(a,t) * \tau)}{\text{Soil}_{\text{water}}(a,t) * \text{Soil}_{\text{depth}} * \rho_{\text{w}}}$	kg N kg $water^{-1}$
19	Total inorganic N lost from mineralized N pool	$N_{\text{loss,IN}}(a,t) = \frac{N_{\text{loss,DIN leached}}(a,t)}{0.8}$	kg N m^{-2} day^{-1}
20	Inorganic N lost from mineralized N pool through gas emissions	$N_{\text{loss,gas}}(a,t) = N_{\text{loss,IN}}(a,t) * 0.2$	kg N m^{-2} day^{-1}

Supplementary Table 4. Summary of model simulations and plant functional type (PFT) composition.

Simulation	PFTs included	Fixation method	Figures
Individual level fixation	Early-, Mid-, Late- successional, Fixer§	Individual	1, 2, 3, S1, S2,S4,S S6,S7,S8
No fixation	Early-, Mid-, Late- successional, Fixer without the fixation ability	No fixation	1, 2, 3, S2,S4
Ecosystem level fixation*	Early-, Mid-, Late- successional, Fixer without the fixation ability§	Ecosystem	2, S1
N saturated**	Early-, Mid-, Late- successional, Fixer§	Individual	S1

§ Initiated at 1.2% of the community composition.

*This simulation has a prescribed quantity of fixed nitrogen that is added directly to the soil on a daily time step. In all other simulations, the fixer employs a facultative strategy and fixed N goes directly to plant.

** This simulation is initiated with high levels of soil N and does not allow for nitrogen loss from the soil.

Supplementary References

1. Medvigy, D., Wofsy, S. C., Munger, J. W., Hollinger, D. Y. & Moorcroft, P. R. Mechanistic scaling of ecosystem function and dynamics in space and time: Ecosystem Demography model version 2. *J. Geophys. Res. Biogeosci.* **114,** G01002 (2009).
2. Medvigy, D., and P. R. Moorcroft. Predicting ecosystem dynamics at regional scales: an evaluation of a terrestrial biosphere model for the forests of northeastern North America. *Philos. Trans. R. Soc. B* **367,** 222-235 (2012).
3. Levine, N. M. *et al.* Ecosystem heterogeneity determines the ecological resilience of the Amazon to climate change. *Proc. Natl. Acad. Sci.* **113,** 793-797 (2016).
4. Longo, M. *et al.* Ecosystem heterogeneity and diversity mitigate Amazon forest resilience to frequent extreme droughts. *New Phytol.* https://doi.org/10.1111/nph.15185 (2018).
5. Kim, Y. *et al.* Seasonal carbon dynamics and water fluxes in an Amazon rainforest. *Glob. Change Biol.* **18,** 1322–1334 (2012).
6. Powell, T. L. *et al.* Confronting model predictions of carbon fluxes with measurements of Amazon forests subjected to experimental drought. *New Phytol.* **200,** 350–365 (2013).
7. Farquhar, G. D., Caemmerer, von, S. V. & Berry, J. A. A biochemical model of photosynthetic CO2 assimilation in leaves of C3 species. *Planta* **149,** 78–90 (1980).
8. Leuning, R., Kelliher, F. M., Pury, D. de & Schulze, E. D. Leaf nitrogen, photosynthesis, conductance and transpiration: scaling from leaves to canopies. *Plant, Cell & Environment* **18,** 1183–1200 (1995).
9. Walko, R. L. et al. Coupled atmosphere-biophysics-hydrology models for environmental modeling. *Journal of applied meteorology* **39,** 931–944 (2000).
10. Moorcroft, P. R., Hurtt, G. C. & Pacala, S. W. A method for scaling vegetation dynamics: the ecosystem demography model (ED). *Ecol. Monogr.* **71,** 557–586 (2001).
11. Batterman, S. A. *et al.* Key role of symbiotic dinitrogen fixation in tropical forest secondary succession. *Nature* **502,** 224–227 (2013).
12. Barron, A. R., Purves, D. W. & Hedin, L. O. Facultative nitrogen fixation by canopy legumes in a lowland tropical forest. *Oecologia* **165**, 511–520 (2011).
13. Hedin L.O., Brookshire, E.N.J., Menge, D.N.L., & Barron, A. The nitrogen paradox in tropical forest ecosystems. *Annu. Rev. Ecol. Evol. Syst.* **40,** 613–35 (2009).

14. Wurzburger, N. & Hedin, L. O. Taxonomic identity determines N_2 fixation by canopy trees across lowland tropical forests. *Ecol Lett* **19,** 62-70, doi:10.1111/ele.12543 (2016).

15. Sheffer, E., Batterman, S. A., Levin, S. A. & Hedin, L. O. Biome-scale nitrogen fixation strategies selected by climatic constraints on nitrogen cycle. *Nature Plants* **1,** 15182, doi:10.1038/nplants.2015.182 (2015).

16. Powers, J. S. & Tiffin, P. Plant functional type classifications in tropical dry forests in Costa Rica: leaf habit versus taxonomic approaches. *Functional Ecology* **24,** 927–936 (2010).

17. Craven, D., Hall, J. S., Berlyn, G. P., Ashton, M. S., & van Breugel, M. Changing gears during succession: shifting functional strategies in young tropical secondary forests. *Oecologia* **179,** 293-305 (2015).

18. Carswell, F. E. et al. Photosynthetic capacity in a central Amazonian rain forest. *Tree Physiology* **20**, 179–186 (2000).

19. Fyllas, N. M. et al. Basin-wide variations in foliar properties of Amazonian forest: phylogeny, soils and climate. *Biogeosciences* **6,** 2677–2708 (2009).

20. Hietz, P. et al. Long-Term Change in the Nitrogen Cycle of Tropical Forests. *Science* **334**, 664–666 (2011).

21. Nardoto, G. B. et al. Understanding the Influences of Spatial Patterns on N Availability Within the Brazilian Amazon Forest. *Ecosystems* **11,** 1234–1246 (2008).

22. Thompson, J. et al. Ecological studies on a lowland evergreen rain forest on Maraca Island, Roraima, Brazil. I. Physical environment, forest structure and leaf chemistry. *Journal of Ecology **80,*** 689–703 (1992).

23. Townsend, A. R., Cleveland, C. C., Asner, G. P. & Bustamante, M. M. Controls over foliar N: P ratios in tropical rain forests. *Ecology* **88,** 107–118 (2007).

24. Millard, P. & Grelet, G. A. Nitrogen storage and remobilization by trees: ecophysiological relevance in a changing world. *Tree Physiology* **30,** 1083–1095 (2010).

25. Gerber, S., Hedin, L. O., Oppenheimer, M., Pacala, S. W. & Shevliakova, E. Nitrogen cycling and feedbacks in a global dynamic land model. *Global Biogeochem. Cycles* **24,**

GB1001 (2010).

26. Gutschick, V. P. Evolved strategies of nitrogen acquisition by plants. *Am. Nat.* **118**, 607–637 (1981).

27. Brzostek, E. R., Fisher, J. B. & Phillips, R. P. Modeling the carbon cost of plant nitrogen acquisition: Mycorrhizal trade-offs and multipath resistance uptake improve predictions of retranslocation. *J. Geophys. Res. Biogeosci*. **119,** 1684–1697 (2014).

28. McGroddy, M. E., Daufresne, T. & Hedin, L. O. Scaling of C: N: P stoichiometry in forests worldwide: implications of terrestrial Redfield-type ratios. *Ecology* **85,** 2390–2401 (2004).

29. Brookshire, E. N., Hedin L. O., Newbold, J. D., Sigman, D. M., & Jackson J. K. Sustained losses of bioavailable nitrogen from montane tropical forests. *Nature Geoscience* **5**,123-126 (2012).

30. Fyllas, N. M. et al. Basin-wide variations in foliar properties of Amazonian forest: phylogeny, soils and climate. *Biogeosciences* **6,** 2677–2708 (2009).

31. Corre, M. D., Veldkamp, E., Arnold, J. & Wright, S. J. Impact of elevated N input on soil N cycling and losses in old-growth lowland and montane forests in Panama. *Ecology* **91,** 1715–1729 (2010).

32. Koehler, B., Corre, M. D., Veldkamp, E., Wullaert, H. & Wright, S. J. Immediate and long-term nitrogen oxide emissions from tropical forest soils exposed to elevated nitrogen input. *Global Change Biology* **15,** 2049–2066 (2009).

33. Oleson, K.W., D.M. et. al. Technical Description of version 4.5 of the Community Land Model (CLM). Ncar Technical Note NCAR/TN-503+STR, National Center for Atmospheric Research, Boulder, CO, 422 pp, (2013).

34. Thornton, P. E. & Rosenbloom, N. A. Ecosystem model spin-up: Estimating steady state conditions in a coupled terrestrial carbon and nitrogen cycle model. *Ecological Modelling* **189,** 25–48 (2005).

35. Xu-Ri & Prentice, I. C. Terrestrial nitrogen cycle simulation with a dynamic global vegetation model. *Global Change Biology* **14,** 1745–1764 (2008).

36. Houlton, B., Sigman, D. & Hedin, L.O. 2006. Isotopic evidence for large gaseous nitrogen losses from tropical rainforests. *Proc. Natl. Acad. Sci.* **103:**8745-8750.

37. Perakis, S. S. & Hedin, L. O. Nitrogen loss from unpolluted South American forests mainly via dissolved organic compounds. *Nature* **415,** 416–419 (2002).

38. Bonn Challenge, www.bonnchallenge.org, accessed 27 June 2017.

39. Poorter, L. *et al.* Biomass resilience of Neotropical secondary forests. *Nature* **530,** 211–214 (2016).

40. Steege, ter, H. *et al.* Continental-scale patterns of canopy tree composition and function across Amazonia. *Nature* **443,** 444–447 (2006).

41. Pan, Y. *et al.* A large and persistent carbon sink in the world's forests. *Science* **333**, 998-993 (2011).

42. Pan, Y., Birdsey R.A., Philips, O.L., & Jackson, R.B. The structure, distribution and biomass of the world's forests *Annu. Rev. Ecol. Evol. Syst.* **44,** 593–622 (2013).

43. Dietze, M. C., S. P. Serbin, C. Davidson, A. R. Desai, X. Feng, R. Kelly, R. Kooper, D. LeBauer, J. Mantooth, and K. McHenry. 2014. A quantitative assessment of a terrestrial biosphere model's data needs across North American biomes. Journal of Geophysical Research: Biogeosciences **119**:286-300.

44. Trugman, A., N. Fenton, Y. Bergeron, X. Xu, L. Welp, and D. Medvigy. 2016. Climate, soil organic layer, and nitrogen jointly drive forest development after fire in the North American boreal zone. Journal of Advances in Modeling Earth Systems 8:1180-1209.

45. Phillips, O. L., T. R. Baker, L. Arroyo, N. Higuchi, T. J. Killeen, W. F. Laurance, S. L. Lewis, J. Lloyd, Y. Malhi, and A. Monteagudo. 2004. Pattern and process in Amazon tree turnover, 1976–2001. Philosophical Transactions of the Royal Society B: Biological Sciences **359**:381-407.
