# Supplementary material for: Tropical carbon sink accelerated by symbiotic dinitrogen fixation
Source: Nat Commun. 2019 Dec 10;10:5637. doi: 10.1038/s41467-019-13656-7 (PMC6904724; doi:10.1038/s41467-019-13656-7)
Supplement: Supplementary file 2 — Reporting Summary [file 41467_2019_13656_MOESM2_ESM.pdf]

nature research

Corresponding author(s): Lars Hedin, Princeton University

Last updated by author(s): Nov 7, 2019

Reporting Summary

Nature Research wishes to improve the reproducibility of the work that we publish. This form provides structure for consistency and transparency in reporting. For further information on Nature Research policies, see Authors & Referees and the Editorial Policy Checklist .

Statistics

For all statistical analyses, confirm that the following items are present in the figure legend, table legend, main text, or Methods section.

n/a	Confirmed	
☐	☒	The exact sample size (n) for each experimental group/condition, given as a discrete number and unit of measurement
☒	☐	A statement on whether measurements were taken from distinct samples or whether the same sample was measured repeatedly
☒	☐	The statistical test(s) used AND whether they are one- or two-sided<br>*Only common tests should be described solely by name; describe more complex techniques in the Methods section.*
☒	☐	A description of all covariates tested
☒	☐	A description of any assumptions or corrections, such as tests of normality and adjustment for multiple comparisons
☒	☐	A full description of the statistical parameters including central tendency (e.g. means) or other basic estimates (e.g. regression coefficient) AND variation (e.g. standard deviation) or associated estimates of uncertainty (e.g. confidence intervals)
☒	☐	For null hypothesis testing, the test statistic (e.g. F, t, r) with confidence intervals, effect sizes, degrees of freedom and P value noted<br>*Give P values as exact values whenever suitable.*
☒	☐	For Bayesian analysis, information on the choice of priors and Markov chain Monte Carlo settings
☒	☐	For hierarchical and complex designs, identification of the appropriate level for tests and full reporting of outcomes
☒	☐	Estimates of effect sizes (e.g. Cohen's d, Pearson's r), indicating how they were calculated

Our web collection on statistics for biologists contains articles on many of the points above.

Software and code

Policy information about availability of computer code

Data collection	n/a
Data analysis	n/a

For manuscripts utilizing custom algorithms or software that are central to the research but not yet described in published literature, software must be made available to editors/reviewers. We strongly encourage code deposition in a community repository (e.g. GitHub). See the Nature Research guidelines for submitting code & software for further information.

Data

Policy information about availability of data

All manuscripts must include a data availability statement. This statement should provide the following information, where applicable:

- Accession codes, unique identifiers, or web links for publicly available datasets
- A list of figures that have associated raw data
- A description of any restrictions on data availability

The code for the ED2 model used in this publication is available in the GitHub repository https://github.com/davidmedvigy/Levy-Varon-etal. The Supplementary Methods provides details on variables and parameters used to create the model. The field data used in Figures 1-3 is available from the corresponding author upon reasonable request.

Field-specific reporting

Please select the one below that is the best fit for your research. If you are not sure, read the appropriate sections before making your selection.

Ecological, evolutionary & environmental sciences study design

All studies must disclose on these points even when the disclosure is negative.

Study description	We evaluate the role of nitrogen fixing trees in forest carbon uptake by combining (i) field observations from 112 plots spanning 300yrs of succession in Panamanian tropical forests, and (ii) a new individual-based model that resolves nitrogen and light competition at the scale of individual trees. The model is based on a development of the ED2 individual-based vegetation model.
Research sample	This is a modeling study and all field data derive from a previous publication (Batterman et al. Nature 502, 224–227, 2013). The dataset derives from 112 plots of community composition from tropical Panamanian rain forests. The data analyzed and approximated by our model is: (i) community composition of tree functional type including nitrogen fixing, early successional, mid-successional and late successional; (ii) properties at the ecosystem level including forest basal area and carbon and nitrogen cycling.
Sampling strategy	This is a modeling study and all field data derive from a previous publication (Batterman et al. Nature 502, 224–227, 2013). The sampling strategy is detailed in Batterman et al and summarized in Methods and Supplemental Methods of this manuscript.
Data collection	This is a modeling study and all field data derive from a previous publication (Batterman et al. Nature 502, 224–227, 2013). The sampling strategy is detailed in Batterman et al and summarized in Methods and Supplemental Methods of this manuscript.
Timing and spatial scale	This is a modeling study and all field data derive from a previous publication (Batterman et al. Nature 502, 224–227, 2013). The sampling strategy is detailed in Batterman et al and summarized in Methods and Supplemental Methods of this manuscript.
Data exclusions	There were no exclusions of any orignial data from Batterman et al. Nature 502, 224–227, 2013.
Reproducibility	This is a modeling study.
Randomization	This is a modeling study.
Blinding	Not applicable

Did the study involve field work? ☐ Yes ☒ No

Reporting for specific materials, systems and methods

We require information from authors about some types of materials, experimental systems and methods used in many studies. Here, indicate whether each material, system or method listed is relevant to your study. If you are not sure if a list item applies to your research, read the appropriate section before selecting a response.

Materials & experimental systems

n/a	Involved in the study	
☒	☐	Antibodies
☒	☐	Eukaryotic cell lines
☒	☐	Palaeontology
☐	☒	Animals and other organisms
☒	☐	Human research participants
☒	☐	Clinical data

Methods

n/a	Involved in the study	
☒	☐	ChIP-seq
☒	☐	Flow cytometry
☒	☐	MRI-based neuroimaging

Animals and other organisms

Policy information about studies involving animals; ARRIVE guidelines recommended for reporting animal research

Laboratory animals	None
Wild animals	This is a modeling study but it includes data from Batterman et al. Nature 502, 224–227, 2013 on wild trees that grow in tropical rain forests of Panama.
Field-collected samples	n/a
Ethics oversight	n/a

Note that full information on the approval of the study protocol must also be provided in the manuscript.
